# Atomic Tuning of Metal‐Support Interactions for Pathway‐Selective CO_2_ Photoreduction on TiO_2_

**DOI:** 10.1002/advs.202521625

**Published:** 2026-01-22

**Authors:** Dongyun Kim, Wonjae Ko, Byoung‐Hoon Lee, Sanghoon Kim, Yun Do Kim, Hyunsoo Ahn, Yoon Jung, Chan Woo Lee, Kug‐Seung Lee, Eunhee Gong, Junho Lee, Minho Kim, Taeghwan Hyeon, Su‐Il In

**Affiliations:** ^1^ Department of Energy Science & Engineering DGIST Daegu Republic of Korea; ^2^ Center For Nanoparticle Research Institute For Basic Science (IBS) Seoul Republic of Korea; ^3^ School of Chemical and Biological Engineering, and Institute of Chemical Processes Seoul National University Seoul Republic of Korea; ^4^ KU‐KIST Graduate School of Converging Science and Technology Korea University Seoul Republic of Korea; ^5^ Department of Integrative Energy Engineering Korea University Seoul Republic of Korea; ^6^ Department of Applied Chemistry Kyung Hee University Yongin Gyeonggi Republic of Korea; ^7^ Pohang Accelerator Laboratory (PAL) Pohang Republic of Korea

**Keywords:** metal‐support interactions, oxygen vacancies, photocatalytic CO_2_ reduction, reaction pathway modulation, single‐atom catalysts

## Abstract

Photocatalytic CO_2_ conversion demands precise control over multielectron reaction pathways to achieve selective solar fuel production. Atomically dispersed Fe and Cu catalysts on TiO_2_ direct divergent photoreduction outcomes—CO via Fe and CH_4_/C_2_H_6_ via Cu—through modulation of CO intermediate binding. Using in situ diffuse reflectance infrared Fourier transform spectroscopy (DRIFTS), X‐ray absorption fine structure (XAFS), and density functional theory (DFT) calculations, this study unveils how metal–support interactions reshape electronic structure, stabilize key intermediates, and create oxygen vacancies that enhance CO_2_ adsorption in the dark. The Cu sites further promote C─C coupling, enabling multicarbon products under mild conditions. The optimized single‐atom catalysts show up to 55.7‐fold enhancement in CO and 44.5‐fold in CH_4_ yields, far surpassing pristine TiO_2_. This work illustrates how rational single‐atom design can precisely manipulate reaction pathways at the atomic scale, offering a unified framework for selective photoredox catalysis in CO_2_ reduction.

## Graphical abstract

Single‐atom Fe and Cu catalysts anchored on TiO_2_ steer photocatalytic CO_2_ reduction toward distinct pathways. Fe sites favor rapid *CO desorption and selective CO formation, whereas Cu sites stabilize *CHO intermediates, enabling deep reduction and C─C coupling. Combined spectroscopy and DFT calculations reveal how metal–support interactions and oxygen vacancies govern pathway selectivity at the atomic scale.

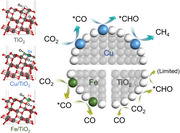

## 1 Introduction

TiO_2_ has long stood as a benchmark material in photocatalysis, praised for its environmental friendliness, cost‐effectiveness, and photostability [1, 2, 3]. Yet, its application in CO_2_ photoreduction continues to fall short of expectations—limited not only by its intrinsic wide bandgap and fast charge recombination [4, 5, 6, 7], but also by a persistent lack of understanding of its low surface catalytic activity [8, 9]. Despite numerous engineering efforts, including cocatalyst deposition [5, 10, 11], structural tuning [5, 6, 8], and band modulation [9, 12], the fundamental origins of TiO_2_’s poor catalytic performance under CO_2_ reduction conditions remain obscure.

At the heart of photocatalytic selectivity and efficiency lie the electronic interactions at the catalyst surface—particularly at the interface between the active site and the support [13, 14, 15]. In this regard, single‐atom catalysts (SACs) offer a compelling opportunity to deconstruct and re‐engineer surface reactivity with atomic precision [16]. By enabling isolated metal sites with tunable coordination environments, SACs not only expand the chemical space beyond that of conventional nanoparticles, but also allow for systematic interrogation of metal‐support electronic interactions that dictate the binding and transformation of key reaction intermediates [17, 18, 19].

Recent studies have highlighted the catalytic potential of SACs across diverse reactions, from plastic upcycling [10, 20, 21], H_2_ evolution [17, 18, 22], and N_2_ reduction [23, 24, 25, 26] to CO_2_ conversion [13, 27, 28, 29, 30, 31]. Yet, the structure–function relationships that govern CO_2_ photoreduction on SAC‐modified TiO_2_ remain largely uncharted [32, 33]. In particular, the interplay between singleatom identity, induced surface defects, and the adsorption and activation of CO_2_‐derived intermediates has not been fully elucidated, representing a critical gap in the rational design of photocatalysts.

To address this gap, we strategically selected Fe and Cu single atoms as a contrasting metal pair that exhibits fundamentally different metal‐support electronic interactions on TiO_2_. Fe^3+^ single atoms anchored at Ti‐vacancy‐related sites impose minimal lattice distortion and favor rapid *CO desorption, whereas Cu^2+^ single atoms induce oxygen‐vacancy formation and stabilize *CO intermediates, enabling deeper hydrogenation toward hydrocarbon products. This designed comparative system, therefore allows mechanistic decoupling of pathway‐selective CO_2_ photoreduction.

In this study, we bridge that gap by integrating rational SAC design, in situ spectroscopic analysis, and first‐principles theory to uncover the molecular‐level mechanisms that govern CO_2_ photoreduction on TiO_2_. We show that pristine TiO_2_ lacks accessible Lewis base sites for CO_2_ adsorption in the absence of illumination. However, anchoring Fe or Cu atoms onto the TiO_2_ surface induces oxygen vacancies (V_O_) that act as both electron‐rich centers and Lewis bases, enabling CO_2_ chemisorption and activation. These metal‐specific perturbations generate distinct reactivity profiles: Fe atoms—favoring high coordination with minimal lattice distortion—enhance CO desorption and afford near‐unity CO selectivity with a >50‐fold boost in CO_2_‐to‐CO productivity. In contrast, Cu atoms—prone to lattice distortion and vacancy formation—stabilize *CO intermediates and promote further reduction to CH_4_ and C_2_H_6_, resulting in a marked increase in hydrocarbon productivity.

Together, these findings offer a design principle for tailoring photocatalytic pathways through precise modulation of interfacial electronic structure at the single‐atom level. Our study provides a chemically intuitive basis for constructing single‐atom oxide interfaces that selectively activate CO_2_ and guide it through energetically favorable multielectron transfer pathways. By illuminating how atomic‐scale interactions steer multielectron CO_2_ reduction chemistry, our work advances both the fundamental understanding and practical design of next‐generation photocatalysts.

## 2 Results and Discussion

### 2.1 Material Synthesis and Characterization

Single‐atom photocatalysts featuring isolated Fe or Cu atoms on TiO_2_ were synthesized via a template‐mediated “wrap–bake–peel” approach, previously established to yield site‐specific SACs by confining metal precursors and guiding them toward thermodynamically favorable Ti vacancy sites [13, 17]. The atomic loading was precisely controlled by varying the amount of metal precursor introduced during synthesis, resulting in a series of materials denoted as *x*Fe/TiO_2_ and *x*Cu/TiO_2_, where *x* represents the metal content in wt.%, as determined by inductively coupled plasma atomic emission spectroscopy (ICP‐AES) (Tables S1 and S2).

**FIGURE 1:**
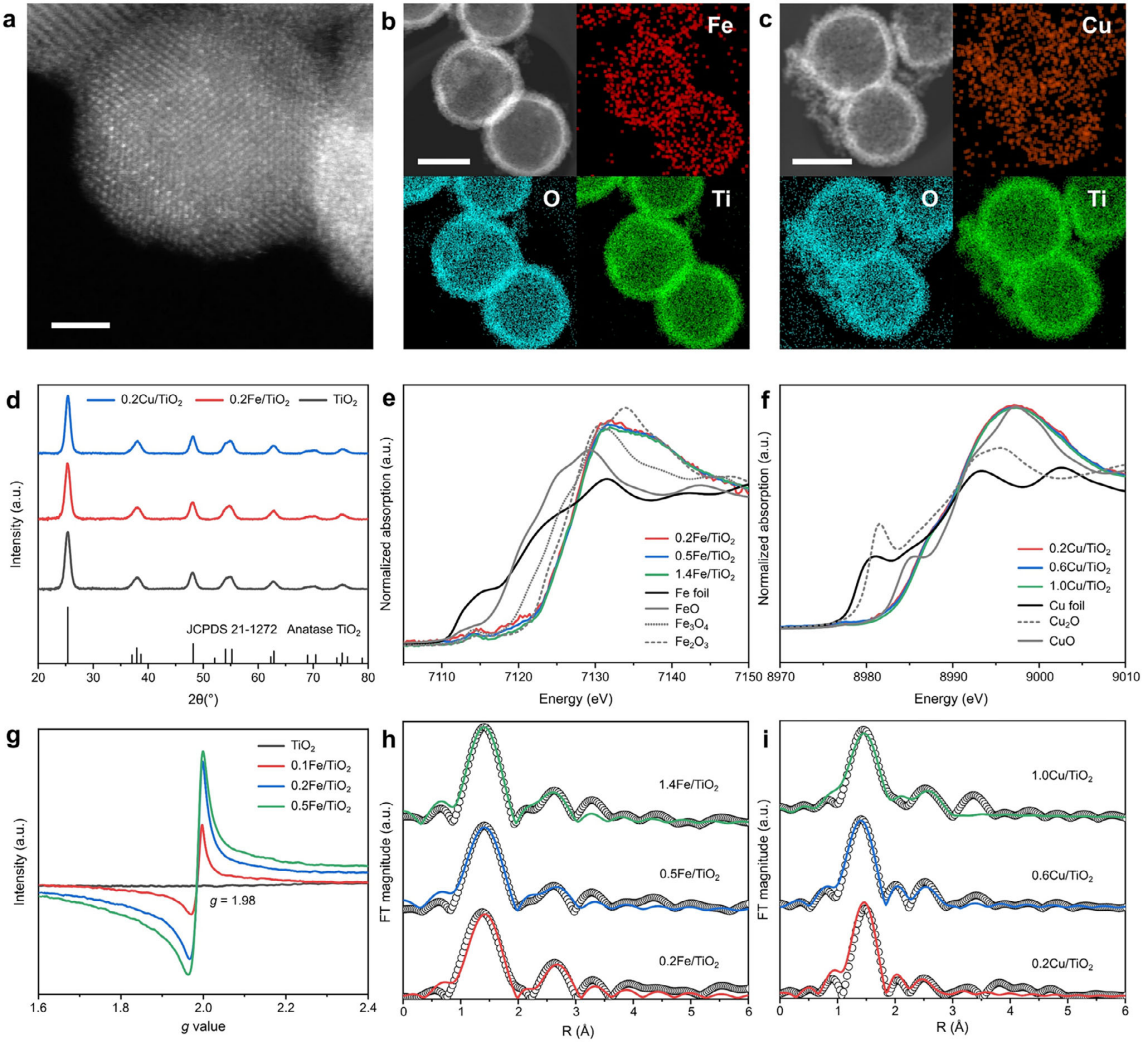
Characterization of Fe/TiO_2_ and Cu/TiO_2_ nanoparticles. (a) HAADF‐STEM image of 0.5Fe/TiO_2_. Scale bar = 2 nm. (b) EDS elemental mapping of 0.5Fe/TiO_2_. Scale bar = 200 nm. (c) EDS elemental mapping of 1.0Cu/TiO_2_. Scale bar = 200 nm. (d) XRD patterns of TiO_2_, 0.2Fe/TiO_2_, and 0.2Cu/TiO_2_. (e) Fe *K*‐edge XANES of 0.2Fe/TiO_2_, 0.5Fe/TiO_2_, 1.4Fe/TiO_2_ and reference samples. (f) Cu *K*‐edge XANES spectra of 0.2Cu/TiO_2_, 0.6Cu/TiO_2_, 1.0Cu/TiO_2_, and reference samples. (g) EPR spectra of TiO_2_, 0.1Fe/TiO_2_, 0.2Fe/TiO_2_, and 0.5Fe/TiO_2_ measured at 150 K. (h) Fe *K*‐edge FT‐EXAFS of 0.2Fe/TiO_2_, 0.5Fe/TiO_2_, 1.4Fe/TiO_2_ (circles), and their fitting results (lines). (i) Cu *K*‐edge FT‐EXAFS of 0.2Cu/TiO_2_, 0.6Cu/TiO_2_, 1.0Cu/TiO_2_ (circles), and their fitting results (lines).

Transmission electron microscopy (TEM) revealed that both Fe/TiO_2_ and Cu/TiO_2_ adopt hollow‐sphere morphologies (Figures S1 and S2), and high‐angle annular dark‐field scanning transmission electron microscopy (HAADF‐STEM) images of representative samples (0.5Fe/TiO_2_ and 0.6Cu/TiO_2_) confirmed the absence of metal aggregation, indicating true atomic dispersion of Fe and Cu (Figure 1a, Figure S3). Energy‐dispersive X‐ray spectroscopy (EDS) elemental mapping further demonstrated the uniform distribution of Fe and Cu atoms across the TiO_2_ matrix (Figure 1b,c, Figures S4 and S5). Powder X‐ray diffraction (XRD) patterns exhibited only the characteristic reflections of anatase TiO_2_, with no detectable secondary phases (Figure 1d, Figures S6 and S7). These observations confirm the successful incorporation of single metal atoms without altering the bulk crystal structure.

To probe the local atomic and electronic environment of the single‐atom sites, we employed X‐ray absorption spectroscopy (XAS) and electron paramagnetic resonance (EPR) spectroscopy. X‐ray absorption near‐edge structure (XANES) analysis at the Fe and Cu K‐edges revealed that both Fe/TiO_2_ and Cu/TiO_2_ samples exhibit identical edge features across varying loadings, indicating well‐defined and reproducible metal speciation. The Fe K‐edge spectra matched closely with Fe_2_O_3_ standards, indicating a Fe^3+^ oxidation state (Figure 1e), while the Cu K‐edge spectra aligned with CuO, supporting the exclusive presence of Cu^2+^ species (Figure 1f). Notably, the absence of pre‐edge features characteristic of metallic Cu or Cu(I) species confirmed the atomic dispersion and oxidation state purity of the Cu centers.

Building on previous findings that Cu atoms on TiO_2_ generate Ti^3+^ states and oxygen vacancies (V_O_) via electronic interaction [13], we examined whether analogous behavior occurs in Fe/TiO_2_. EPR measurements revealed strong Ti^3+^ signals at g = 1.98 in all Fe/TiO_2_ samples [10, 34, 35], with signal intensity increasing proportionally to Fe loading (Figure 1g), indicating that Fe incorporation also induces Ti^3+^ formation through metal‐support charge transfer. In Cu/TiO_2_, overlapping EPR signals from Cu^2+^ and Ti^3+^ were observed (Figure S8) [36], further supporting their electronic interaction with the support. To further probe the surface chemical environment of the optimized catalysts, XPS measurements were performed as a complementary surface‐sensitive technique to EPR and XAFS analyses, providing additional information on the surface valence features and local electronic environment of Fe, Cu, and lattice oxygen species (Figure S9) [37, 38].

To elucidate the atomic structure of the metal centers, extended X‐ray absorption fine structure (EXAFS) analysis was performed at both Fe and Cu K‐edges. Fourier‐transformed EXAFS spectra consistently exhibited two dominant scattering paths corresponding to M─O and M─Ti (M═Fe or Cu) coordination environments (Figure 1h,i). Quantitative fitting revealed that both Fe and Cu atoms locate Ti vacancy sites on the TiO_2_ (Figure 1h,i, Figures S10 and S11, Tables S3 and S4). Therefore, we confirmed that the prepared Fe/TiO_2_ and Cu/TiO_2_ photocatalysts have single metal atoms in Ti vacancies on TiO_2_, which enrich Ti^3+^ states on TiO_2_ surfaces by the electronic interaction. Importantly, Cu atoms exhibited lower coordination numbers in both M─O and M─Ti shells compared to Fe, indicative of a more flexible and undercoordinated environment. These structural differences are consistent with theoretical predictions and correlate with the distinct reactivity profiles observed under CO_2_ photoreduction conditions.

Together, these findings demonstrate the successful synthesis of structurally well‐defined SACs with atomically dispersed Fe and Cu atoms stabilized in Ti vacancies of TiO_2_. The distinct local coordination environments and associated electronic interactions of Fe and Cu lay the foundation for understanding their divergent catalytic roles in modulating CO_2_ photoreduction pathways, as discussed in the following sections.

To assess the structural stability of the single‐atom catalysts under photocatalytic conditions, post‐reaction EPR and XAS measurements were performed for the optimized 0.5Fe/TiO_2_ and 0.2Cu/TiO_2_ samples following photocatalytic stability testing. As shown in Figure S12, the EPR spectra of both Fe/TiO_2_ and Cu/TiO_2_ after reaction retain the characteristic Ti^3+^ signal at g = 1.98, with no emergence of additional features attributable to metallic species or agglomeration. The preservation of the g‐value and overall spectral profile indicates that the local electronic environment induced by single‐atom incorporation remains largely unchanged during photocatalysis.

Consistently, XANES spectra collected after reaction show no discernible edge shift or change in spectral shape compared to the fresh catalysts, indicating that the oxidation states of Fe and Cu are preserved (Figure S13). Moreover, the Fourier‐transformed EXAFS spectra exhibit nearly identical M─O and M─Ti coordination features before and after reaction, with no appearance of M–M scattering contributions. Together, the post‐reaction spectroscopic analyses confirm the structural robustness of the atomically dispersed Fe and Cu sites under illumination, excluding significant metal migration or aggregation during photocatalytic operation.

### 2.2 Photocatalytic CO_2_ Reduction Experiment

The photocatalytic reduction of CO_2_ was carried out in a gas–solid phase system under 1 Sun illumination using a Xenon lamp, without any sacrificial agents. Under these conditions, single‐atom TiO_2_ photocatalysts incorporating either Fe or Cu exhibited markedly enhanced CO_2_ conversion compared to pristine TiO_2_ (Table S5). Notably, negligible H_2_ evolution was detected under the gas–solid reaction conditions, indicating effective suppression of the hydrogen evolution reaction due to limited proton availability from vapor‐phase H_2_O (Figure S14a).

**FIGURE 2:**
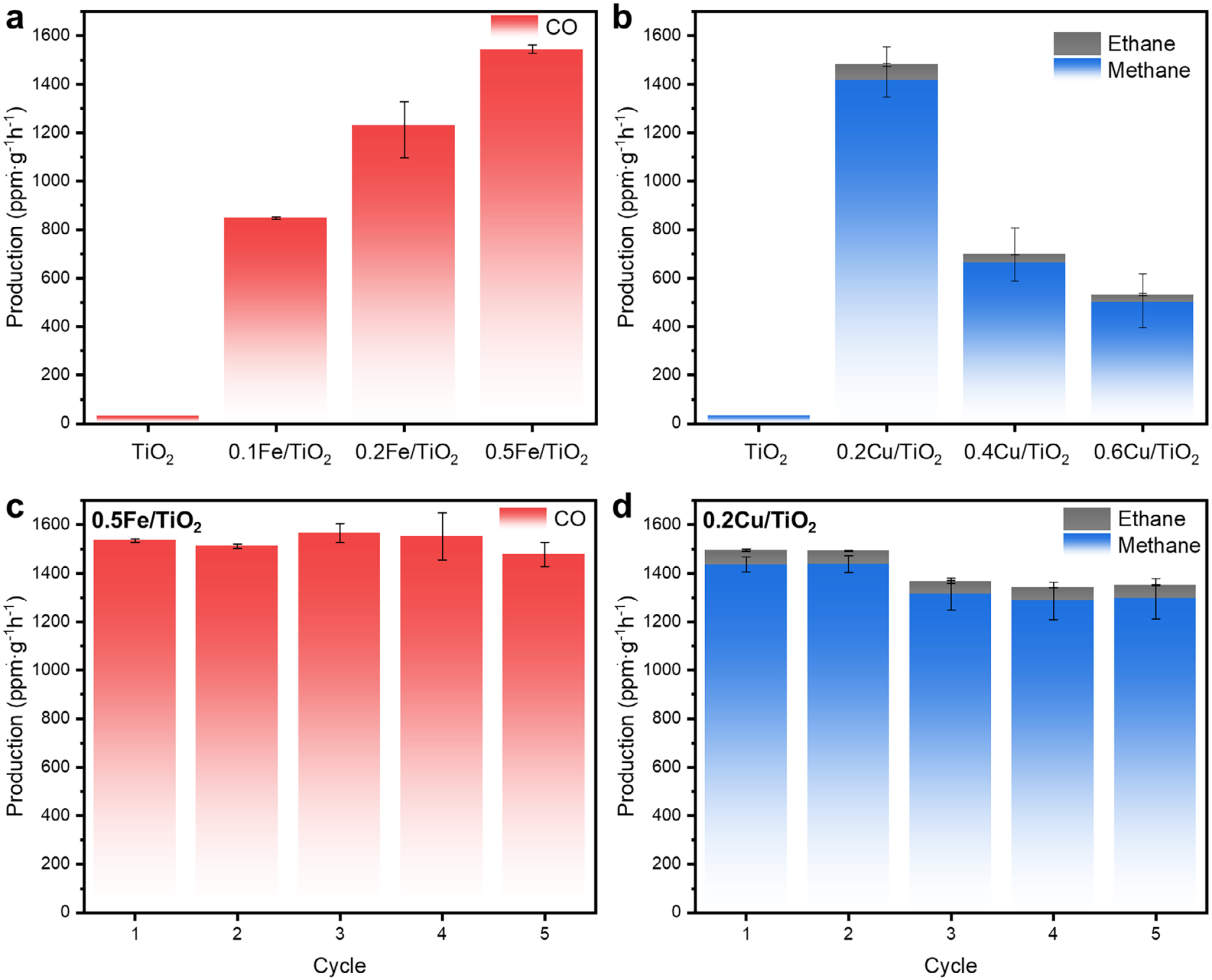
Photocatalytic CO_2_ reduction performance of (a) TiO_2_, 0.1Fe/TiO_2_, 0.2Fe/TiO_2_, 0.5Fe/TiO_2_ and (b) TiO_2_, 0.2Cu/TiO_2_, 0.4Cu/TiO_2_, 0.6Cu/TiO_2_. Cyclic stability of (c) 0.5Fe/TiO_2_ and (d) 0.2Cu/TiO_2_.

Specifically, bare TiO_2_ produced CO at a modest rate of 28.06 ppm g^−1^ h^−1^, whereas 0.5Fe/TiO_2_ achieved a 55.7‐fold increase, reaching 1562.5 ppm g^−1^ h^−1^ (Figure 2a). The CO production rate showed a positive correlation with Fe loading, indicating that Fe incorporation promotes both adsorption and efficient conversion of CO_2_ to CO. In contrast, Cu‐containing SACs shifted the product selectivity from CO to hydrocarbons, predominantly CH_4_ and C_2_H_6_ (Figure 2b, Figure S14b). While bare TiO_2_ generated CH_4_ at a rate of 31.83 ppm g^−1^ h^−1^, 0.2Cu/TiO_2_ achieved a 44.5‐fold enhancement, producing 1416.9 ppm g^−1^ h^−1^ of CH_4_ along with 64.19 ppm g^−1^ h^−1^ of C_2_H_6_. Notably, increasing Cu content beyond 0.2 wt.% led to a decrease in hydrocarbon productivity, suggesting a non‐linear structure–function relationship tied to atomic dispersion and surface dynamics. In the stability tests, 0.5Fe/TiO_2_ maintained nearly constant CO production over five cycles (Figure 2c). While 0.2Cu/TiO_2_ also exhibited cyclic stability for five cycles with only 7% decline after the third cycle, indicating that the Cu active sites also retained superior stability (Figure 2d).

These results reveal several mechanistic insights into the behavior of single‐atom modified TiO_2_ photocatalysts. First, the incorporation of Fe or Cu single atoms distinctly alters the CO_2_ reduction pathway: Fe promotes selective formation of CO, whereas Cu favors multielectron reduction leading to hydrocarbon formation. Second, Fe loading enhances both selectivity and rate of CO formation, consistent with its role in facilitating *CO desorption and suppressing further hydrogenation. Conversely, the decline in hydrocarbon productivity with higher Cu content suggests over‐saturation or clustering effects that attenuate the desired electronic interactions.

We attribute these divergent catalytic behaviors to differences in the coordination geometry and electronic structure of Fe and Cu centers within the TiO_2_ lattice. These metal‐support interactions directly impact the stabilization of reaction intermediates, charge transfer dynamics, and ultimately the selectivity of CO_2_ reduction products. Together, these findings underscore the power of single‐atom interface engineering in tailoring photoreduction pathways and open a chemically tractable route to modulate multielectron CO_2_ conversion with atomic precision.

To uncover the origins of product selectivity and activity in single‐atom photocatalysts, we investigated the relationship between surface structure and CO_2_ adsorption behavior using temperature‐programmed desorption (CO_2_‐TPD) and in situ diffuse reflectance infrared Fourier transform (DRIFT) spectroscopy. Bare TiO_2_ showed negligible CO_2_ adsorption in the dark (Figure S15a) [10, 39, 40], consistent with its known lack of oxygen vacancies (V_O_) and Ti^3+^ sites [41, 42], which are critical for chemisorption under non‐irradiated conditions. It should be noted that the ‘dark’ adsorption behavior originates purely from vacancy‐induced Lewis basic sites, rather than from any changes in optical absorption properties. In situ DRIFT analysis during CO_2_ photoreduction revealed the gradual appearance of a band at 1235 and 1412 cm^−1^, corresponding to HCO_3_^−^ species, and a signal at 1712 cm^−1^ attributed to the *CHO intermediate (Figure S15b) [41, 43, 44]. The accumulation of *CHO suggests that while CO_2_ is partially reduced under illumination [41, 45, 46], the full conversion to CH_4_ remains kinetically hindered due to inefficient charge and proton transfer on bare TiO_2_ [47, 48, 49].

**FIGURE 3:**
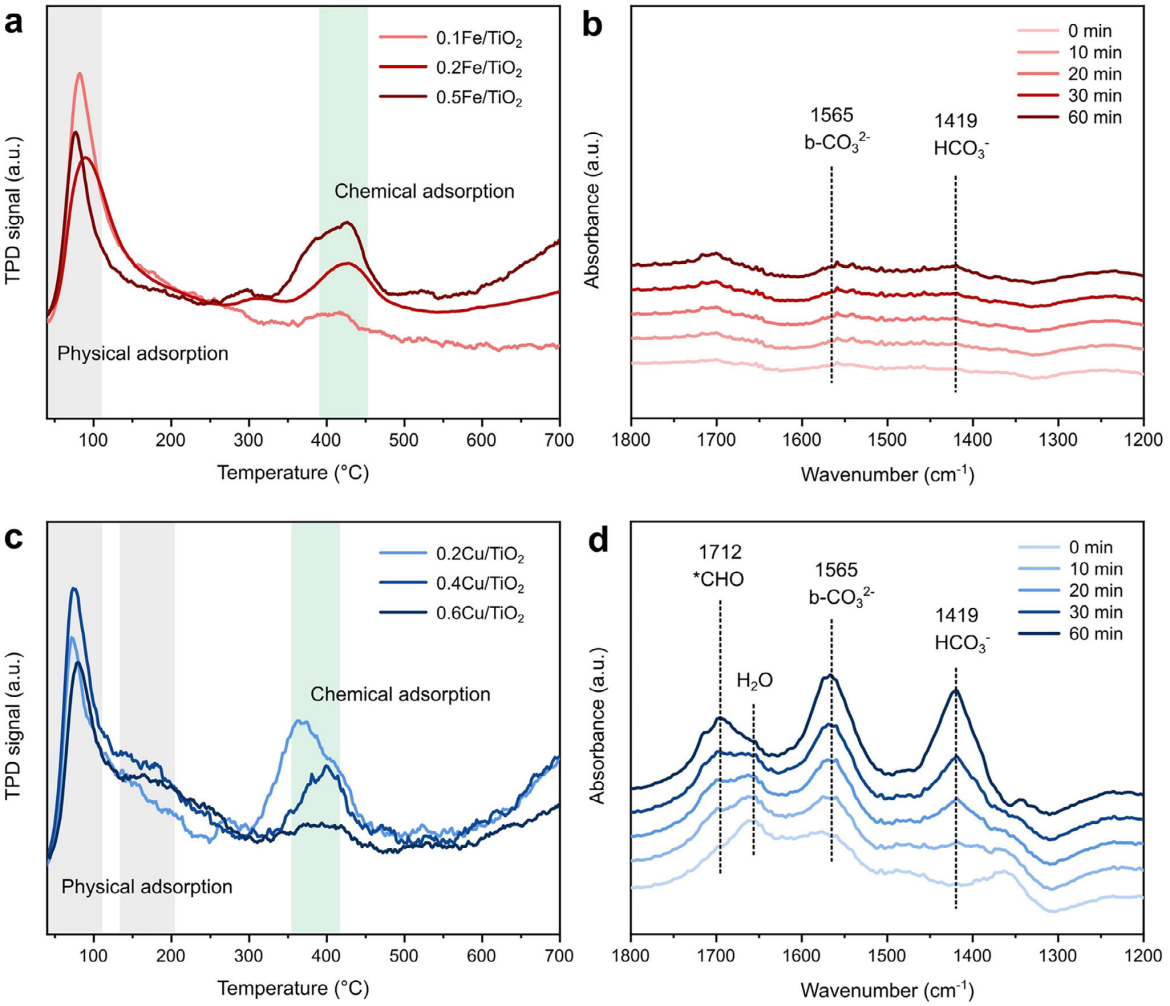
(a) TPD analysis of 0.1Fe/TiO_2_, 0.3Fe/TiO_2_, and 0.5Fe/TiO_2_ and (b) in situ DRIFT analysis of 0.5Fe/TiO_2_. (c) TPD analysis of 0.2Cu/TiO_2_, 0.4Cu/TiO_2_, and 0.6Cu/TiO_2_ and (d) in situ DRIFT analysis of 0.2Cu/TiO_2_.

By contrast, Fe single‐atom incorporation into TiO_2_ markedly altered CO_2_ adsorption behavior. CO_2_‐TPD revealed a chemisorption peak near 400°C, the intensity of which increased with Fe loading (Figure 3a), indicative of enhanced binding strength at Fe‐modified sites. Importantly, this enhancement was attributed to the formation of Fe^3+^ centers—confirmed by X‐ray absorption spectroscopy (XAFS)—which are isovalently substituted for Ti^4+^ within the lattice with minimal local distortion. These Fe^3+^ sites provide Lewis base centers capable of chemisorbing CO_2_ even in the absence of light [50, 51]. In situ DRIFT spectra supported this behavior: at low Fe content (0.1Fe/TiO_2_), bands at 1419 cm^−1^ (HCO_3_^−^) and 1565 cm^−1^ (bidentate CO_3_^2−^) were observed (Figure S16a) [52], but their intensity decreased with increasing Fe concentration (Figure S16b), suggesting that CO_2_ adsorbed onto Fe^3+^ sites is more readily reduced and desorbed at higher Fe loadings (Figure 3b). Furthermore, distinct peaks corresponding to C–H stretching vibrations were not observed for 0.5Fe/TiO_2_ (Figure S17a) [53]. Although CO_2_ adsorption initially proceeded sufficiently, the adsorption rate gradually lagged behind the reaction rate during continuous operation [54]. This efficient *CO desorption prevents *CHO accumulation, explaining the observed high CO selectivity in Fe/TiO_2_. The weak CO binding on Fe^3+^ sites facilitates its desorption before further hydrogenation can occur [55], effectively truncating the reaction pathway at the two‐electron reduction stage.

In contrast, Cu single‐atom sites exhibited a different structure–function profile. CO_2_ chemisorption decreased with increasing Cu loading (Figure 3c), indicating an optimal concentration window for catalytic efficiency. This trend is attributed to local Cu–Cu proximity effects at higher loadings, which disrupt charge localization at isolated Cu single‐atom sites and suppress CO_2_ activation [13, 56]. At low loading, Cu atoms generate strong Lewis base sites, facilitating CO_2_ adsorption through localized electron donation. In situ DRIFT spectroscopy confirmed this: Cu‐decorated TiO_2_ showed stronger HCO_3_^−^ and b‐CO_3_^2−^ signals compared to bare TiO_2_ (Figure 3d), reflecting increased density of surface basic sites. The overall amount of adsorbed CO_2_ remained high, and the adsorption process proceeded more rapidly on Cu/TiO_2_ than on Fe/TiO_2_ [54]. Pronounced OH stretching bands were also observed, indicating the presence of abundant surface hydroxyl groups. In addition, distinct peaks appeared in the range of 3000–2820 cm^−1^, which are attributed to C–H stretching vibrations involving CH_2_/CH_3_‐related intermediates (Figure S17b) [53]. The formation of hydrogenated carbon species (*CH_x_) is regarded as a critical prerequisite for C─C bond formation in CO_2_ reduction, as these intermediates can participate in CO_2_ insertion or coupling pathways leading to C_2_ products [57]. Although a surface‐bound *C_2_ intermediate is not directly observed in the present system, the detection of *CH_x_ species by DRIFTS together with the formation of C_2_H_6_ as a gas‐phase product supports the involvement of Cu‐enabled reaction pathways that permit C─C bond formation. The carbonate signals peaked at 0.2Cu/TiO_2_ and diminished at higher Cu content (0.6Cu/TiO_2_), while the concurrent rise of H_2_O bands (1644 cm^−1^) suggested proton competition or hydroxyl displacement (Figure S18a,b).

Importantly, Cu SACs enable complete photoreduction of the *CHO intermediate to CH_4_ and C_2_H_6_, in contrast to bare TiO_2,_ which accumulates *CHO due to limited photogenerated charge utilization. Theoretical and experimental studies suggest that Cu single atoms induce the spontaneous formation of V_O_ around the metal center by modulating the electronic configuration of the TiO_2_ lattice. This occurs via the emergence of mid‐gap O‐derived states above the Fermi level and symmetry breaking in the local coordination environment [13]. These changes promote dynamic charge redistribution and stabilize key intermediates, facilitating multielectron reduction pathways.

Together, these findings demonstrate how atomic‐scale metal‐support interactions critically govern CO_2_ chemisorption behavior and subsequent reduction dynamics. Whereas Fe^3+^ sites favor rapid *CO desorption and selective CO formation, Cu^2+^ centers enable deeper reduction through intermediate stabilization, highlighting a tunable mechanism to control product distribution in single‐atom photocatalysis (Figure S19).

### 2.3 CO_2_ Photoreduction Mechanism

**FIGURE 4:**
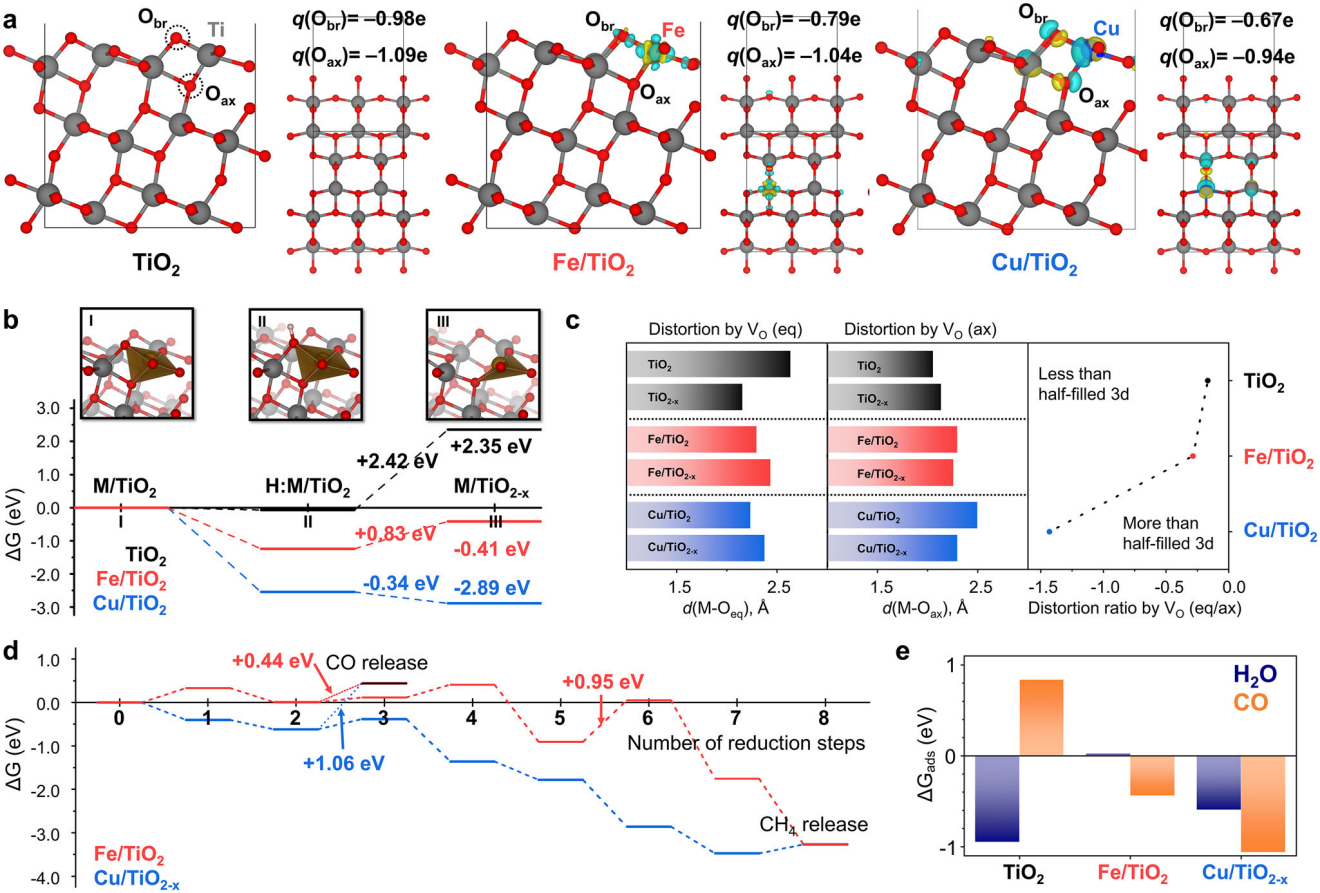
(a) Geometry of DFT optimized structures of TiO_2_, Fe/TiO_2_, and Cu/TiO_2_ (111) surface viewed along the [010] (left) and [100] (right) directions with the charge density difference from TiO_2_, where yellow and cyan indicate electron accumulation and depletion, respectively (isovalue = 0.02 e Å^−3^). The Bader charge of the axial O atom, *q*(O_ax_), and the bridge O atom are to be defected by the O vacancy (V_O_) formation, *q*(O_br_). The atoms are colored in red for O, gray for Ti, brown for Fe, and blue for Cu, respectively. (b) DFT thermodynamics of V_O_ formation in TiO_2_ (black), Fe/TiO_2_ (red), and Cu/TiO_2_ (blue). (c) Quantitative changes in the bond lengths (in Å) of (left) the equatorial and (middle) axial O atoms with the single‐metal atoms, and their ratio, Δ*d*(M‐O_eq_)/Δ*d*(M‐O_ax_). d) DFT thermodynamics of CO_2_ reduction to CO and CH_4_ in Fe/TiO_2_ (red) and Cu/TiO_2‐x_ (blue). (e) Free energy change of adsorption of H_2_O (navy) and CO (orange) at a single‐atom catalyst in TiO_2_, Fe/TiO_2,_ and Cu/TiO_2‐x_.

To elucidate the origin of divergent product selectivity in TiO_2_‐supported single‐atom photocatalysts, we performed density functional theory (DFT) calculations to probe the thermodynamics and electronic structure of active sites on Cu/TiO_2_, Fe/TiO_2_, and bare TiO_2_ (Figure 4a, Figure S20). Particular focus was placed on the formation and role of oxygen vacancies (V_O_), which serve as electron‐rich CO_2_ binding pockets and are widely regarded as key facilitators of photocatalytic reactivity [13].

Our calculations reveal that Cu incorporation substantially lowers the vacancy formation energy, making V_O_ generation highly exergonic on Cu/TiO_2_ (−2.89 eV) compared to Fe/TiO_2_ (−0.41 eV) and pristine TiO_2_ (+2.35 eV) (Figure 4b). This trend is supported by Bader charge analysis of surface bridging oxygen atoms (O_br_), which shows less negative charge in Cu/TiO_2_ (−0.67e) relative to Fe/TiO_2_ (−0.79e) and TiO_2_ (−0.98e), indicating a higher oxidative potential that renders the lattice oxygen more labile (Figure 4a).

Structurally, the enhanced V_O_ formation in Cu/TiO_2_ can be attributed to pronounced axial distortion within the Cu─O octahedron. The axial Cu─O bond length extends to 2.00 Å, longer than those in Fe/TiO_2_ (1.90 Å) and TiO_2_ (1.78 Å), reflecting strong metal‐support interaction and the occupation of $d_{z^{2}}$ orbitals (Figure S21). Comparative analysis of distortion modes further shows that Cu—being a late transition metal with higher d‐electron occupancy (Figure S22)—induces greater axial compression and destabilizes local lattice symmetry more significantly than Fe or Ti (Figure 4c). This distortion not only activates adjacent lattice oxygen atoms toward vacancy formation but also enhances the Lewis basicity of the site, favoring CO_2_ chemisorption. This distortion modulates the frontier orbitals of the active site, lowering the energy barrier for C–C coupling and enabling hydrocarbon formation—a reactivity feature rarely observed in oxide‐supported SACs. These findings align well with experimental EXAFS results, which indicate lower coordination numbers for Cu compared to Fe, consistent with more flexible local environments.

The catalytic implications of V_O_ generation were further investigated through DFT‐derived reaction energetics for CO_2_ reduction. In Cu/TiO_2‐x_, V_O_‐rich environments stabilize CO_2_‐derived intermediates on single Cu atoms and significantly increase the energy required for CO desorption (+1.06 eV), thereby disfavoring premature CO release and enabling deeper hydrogenation toward CH_4_ (Figure 4d). In contrast, Fe/TiO_2_ facilitates *CO desorption with a much lower energy barrier (+0.44 eV), consistent with its high CO selectivity observed experimentally. Moreover, Fe/TiO_2_ exhibits a large energy barrier (+0.95 eV) for the cleavage of the C─O bond in *CH_2_OH, effectively blocking CH_4_ formation.

Finally, we assessed the adsorption free energies of H_2_O and CO—critical descriptors for proton‐coupled electron transfer and intermediate stabilization—in the three systems (Figure 4e). Cu/TiO_2‐x_ exhibited both strong H_2_O and CO binding energies, enabling complete CO_2_ reduction to CH_4_. Fe/TiO_2_ showed weaker interactions, favoring CO release, while pristine TiO_2_, which lacks both V_O_ and efficient electron–proton transfer sites, exhibited highly endergonic CO binding, correlating with its poor catalytic activity.

Together, these theoretical results provide a unified mechanistic framework that links metal–support interactions, local structural distortion, and intermediate stabilization to product selectivity in single‐atom photocatalysis. They further underscore the critical role of electronic perturbation and V_O_ formation in enabling deep CO_2_ reduction on Cu/TiO_2_ SACs.

## 3 Conclusion

We have established a mechanistic framework that elucidates how atomic‐scale metal–support electronic interactions govern CO_2_ adsorption behavior and steer product selectivity during photocatalytic reduction on TiO_2_. Through a combination of experimental and theoretical approaches, we demonstrate that single‐atom incorporation of Fe and Cu into the TiO_2_ lattice induces distinct structural and electronic modifications that fundamentally reshape the CO_2_ activation landscape.

Fe atoms, which substitute Ti^4+^ sites with minimal lattice distortion, promote the formation of Lewis basic sites that enable CO_2_ chemisorption even under dark conditions. The weak *CO binding energy on Fe/TiO_2_ facilitates rapid intermediate desorption, yielding near‐unity selectivity toward CO evolution. In contrast, Cu single atoms enhance local metal–oxygen bond polarization and promote spontaneous oxygen vacancy formation via strong metal–support interactions. These vacancies, in conjunction with the electronic flexibility of Cu^2+^ centers, stabilize CO_2_‐derived intermediates and favor multielectron reduction pathways leading to CH_4_ and C_2_H_6_.

The optimized Fe/TiO_2_ and Cu/TiO_2_ photocatalysts achieved 55.7‐ and 44.5‐fold enhancements in CO_2_ reduction activity, respectively, compared to bare TiO_2_. These results collectively point to a rational design principle: that by precisely tuning metal–support electronic interactions at the atomic level, one can systematically modulate intermediate binding, electron transfer characteristics, and product distribution in oxide‐based photocatalysts. This work offers both mechanistic insight and a broadly applicable strategy for advancing selective CO_2_ conversion through single‐atom interface engineering. The electronic tuning strategies presented here may be broadly applicable to other oxide–metal systems for the selective activation of small molecules, paving the way for programmable photocatalytic platforms in solar fuels research.

## 4 Experimental Section/Methods

### 4.1 Chemicals

Iron (III) chloride hexahydrate (FeCl_3_·6H_2_O), copper (II) chloride dihydrate (CuCl_2_·2H_2_O), tetraethyl orthosilicate (TEOS), and polyvinylpyrrolidone (PVP) (Mw ∼ 55 000) were purchased from Sigma Aldrich. Titanium(IV) n‐butoxide was purchased from Strem Chemicals. Acetonitrile, anhydrous ethyl alcohol (99.9%), sodium hydroxide (NaOH), and ammonia solution (28–30 wt.%) were purchased from Samchun Chemical. All reagents were used as received without further purification. Ultrapure de‐ionized (DI) water (Milli‐Q grade, 18.2 MΩ·cm resistivity, TOC level < 2 ppb) was directly supplied from Milli‐Q water purification system.

### 4.2 Synthesis of Fe/TiO_2_ and Cu/TiO_2_

Fe/TiO_2_ and Cu/TiO_2_ photocatalysts were synthesized by a template‐mediated wrap‐bake‐peel process based on our previous reports (Figure S23) [13, 17]. First, SiO_2_ template nanoparticles were synthesized by sol‐gel reaction. The reaction was preceded by adding 0.86 mL of TEOS into a mixture solution of 4.3 mL H_2_O, 23 mL of ethyl alcohol, and 0.6 mL of aqueous ammonia (28–30 wt.%). After 6 h of reaction with magnetic stirring, the synthesized SiO_2_ nanoparticles were collected by centrifuge, washed two times with ethyl alcohol, and redispersed in 40 mL of anhydrous ethanol. Then, 14 mL of acetonitrile and 0.4 mL of aqueous ammonia (28–30 wt.%) were added to the SiO_2_ solution (solution A). Another solution (solution B, titanium(IV) n‐butoxide solution) was prepared by adding 0.8 mL of titanium(IV) n‐butoxide into the mixture of acetonitrile (2 mL) and anhydrous ethanol (6 mL). Solution B was added rapidly to solution A and stirred for 3 h for TiO_2_ coating on SiO_2_ nanoparticles. The product (SiO_2_@TiO_2_) was centrifuged, washed with ethanol and water, and dispersed in 40 mL of H_2_O. Next, an aqueous solution of 4 mg mL^−1^ FeCl_3_·6H_2_O or CuCl_2_·2H_2_O was added to the SiO_2_@TiO_2_ solution with vigorous stirring (for the volume of metal precursor solution required for each metal loading amount, see Tables S1 and S2). After stirring for 3 h at room temperature, the resulting product was centrifuged, washed with water, and dispersed in 40 mL of water. Into the resulting solution, 400 mg of PVP (Mw ∼ 55 000) was added to adsorb PVP on the surface of nanoparticles, and was stirred overnight. After the PVP adsorption, the solution was centrifuged and redispersed in the mixture of 46 mL of ethanol and 8.6 mL of H_2_O with 10 min of sonication. Then, 1.2 mL of aqueous ammonia (28–30 wt.%) and 1.6 mL of TEOS were added to the solution to wrap SiO_2_@TiO_2_ nanoparticles with SiO_2_ shells. After 4 h, the resulting product was washed, centrifuged, and dried in an 80°C oven overnight. The resulting powder was calcined at 950°C for 2 h in ambient air. After the calcination, SiO_2_ templates were etched with 0.5 M NaOH solution at 90°C for 6 h. The resulting product (Fe/TiO_2_ or Cu/TiO_2_) was centrifuged, washed with water and ethanol, and dried in an 80°C oven overnight. Pristine TiO_2_ was prepared by the same method without the metal precursor adsorption step.

### 4.3 Material Characterization

Transmission electron microscopy (TEM) images were obtained with a JEOL JEM‐2100F (JEOL) microscope at an acceleration voltage of 200 kV. Energy dispersive X‐ray spectroscopy (EDS) was performed with a single drift detector (X‐MaxN, Oxford Instruments). Aberration‐corrected high‐angle annular dark‐field scanning transmission electron microscopy (HAADF‐STEM) images were acquired with JEM ARM‐200F (JEOL) at an acceleration voltage of 200 kV. Inductively coupled plasma‐atomic emission spectroscopy (ICP‐AES) was performed with an Agilent 5100. X‐ray diffraction (XRD) patterns were obtained with a Rigaku SmartLab diffractometer (Cu Kα radiation). X‐ray photoelectron spectroscopy (XPS) measurements were carried out on a Thermo Scientific K‐alpha photoelectron spectrometer using Al Kα radiation as the X‐ray source. Electron paramagnetic resonance spectroscopy (EPR) measurements were performed with an EMXmicro system (Bruker) at 150 K. X‐ray absorption spectroscopy (XAS) was conducted at 8C nano‐probe XAFS beamline (BL8C) of Pohang Light Source (PLS‐II) in the 3.0 GeV storage ring, with a ring current of 250 mA. Energy calibration was performed using reference foils before the measurements. The obtained spectra were processed with Demeter software. Model fittings for extended X‐ray absorption fine structure (EXAFS) were performed with the ARTEMIS program, using a Fourier transform range of 3–9 Å^−1^ for Fe *K*‐edge and 3–10 Å^−1^ for Cu *K*‐edge, with a Hanning window applied between 1.25 and 3 Å. Temperature programmed desorption (TPD) analysis was performed with AutoChem II (micromeritics). In the TPD experiment, CO_2_ was adsorbed onto the catalyst for 30 min, followed by temperature ramping under He flow for analysis.

### 4.4 Photochemical Experiments

The photocatalytic CO_2_ reduction with H_2_O using the as‐prepared materials was conducted in a batch‐type reactor system (Figure S24). A 40 mg sample was placed in a 15.4 mL stainless steel reactor, sealed with a quartz window. CO_2_ (1000 ppm) passed through a water bubbler into the reactor, which was purged with moist CO_2_ (3 times). The reactor was filled with wet CO_2_ and irradiated with simulated sunlight (1 sun, AM 1.5 G filter). After one hour, a 500 µL sample was analyzed using a Shimadzu GC‐2014 with a thermal conductivity detector (TCD, Restek RT Msieve 5A column, ID = 0.53 mm, length = 30 m) and a flame ionization detector (FID, Restek Rt‐Q‐bond column, ID = 0.53 mm and length = 30 m) with helium as carrier gas.

### 4.5 DRIFT Analysis

Diffuse reflectance Fourier transform spectroscopy (DRIFTS) was employed to analyze intermediates during the reaction (Figure S25). In situ DRIFT spectra were recorded using a Nicolet iS50 (Thermo Fisher Scientific) spectrometer, Praying Mantis Reaction Chambers (HVC‐DRM), and HgCdTe (MCT) detector. The samples were pretreated in a vacuum at 100°C and then cooled to room temperature prior to measurement. They were subsequently exposed to CO_2_ and H_2_O for 30 min, followed by light irradiation to collect the data. Measurements were taken in the dark and at intervals after Xe lamp illumination. The spectra were obtained with 32 scans at a resolution of 4 cm^−1^.

### 4.6 Theoretical Simulation

Density functional theory calculation performed using Vienna ab initio simulation package (VASP) 5.4.1 [58, 59, 60, 61], with pseudopotentials generated by the projector augmented wave (PAW) method [62]. The Perdew‐Burke‐Ernzerhof (PBE) [63] functional was chosen for exchange‐correlation approximation, and Grimme's D3 method with a Becke‐Johnson damping function [64] was employed for van der Waals correction. The kinetic energy cutoff for plane waves was set as 450 eV. Structures were constructed by using anatase (101) surface with a vacuum slab of 16 Å, and a Monkhorst‐Pack (3 × 4 × 1)*k*‐point grid was adopted for slab models. They were optimized until the final energy difference became 0.1 meV, while the bottom two layers were fixed. Molecules were optimized in a 30 × 30 × 30 Å^3^ vacuum box. Vibrational frequency was calculated through a finite difference method to determine vibrational entropy. All of the structures were visualized using VESTA 3.5.7 [65]. As in a previous work [13], the chemical potential of a proton‐electron pair at 0 V was determined by a half value of H_2(g),_ with the potential of a photoexcited electron referenced to the conduction band minimum (CBM) of TiO_2_, −0.25V_RHE_ [66].

## Author Contributions

D.K., W.K., B.H.L., M.K., T.H., and S.I.I. conceived the research. D.K., W.K., B.H.L., E.G., and M.K. designed the experiments. W.K., B.H.L., Y.D.K., H.A., Y.J., and C.W.L. performed and analyzed the material synthesis. D.Y. and E.G. performed photochemical measurements. S.K. and M.K. performed computational analysis. K.S.L. conducted the XAFS analysis. E.G. and J.L. performed DRIFT analysis. D.K., W.K., B.H.L., M.K., T.H., and S.I.I. wrote the manuscript. M.K., T.H., and S.I.I. supervised the project. All the authors commented on the manuscript.

## Conflicts of Interest

The authors declare no conflicts of interest.

## Supporting information




**Supporting File 1**: advs73950‐sup‐0001‐SuppMat.docx.

## Data Availability

The data that support the findings of this study are available in the supplementary material of this article.
