# Supplementary material for: Atomic Tuning of Metal‐Support Interactions for Pathway‐Selective CO2 Photoreduction on TiO2
Source: Adv Sci (Weinh). 2026 Jan 22:e21625. Online ahead of print. doi: 10.1002/advs.202521625 (PMC13325499; doi:10.1002/advs.202521625)
Supplement: Supplementary file 1 — Supporting File 1: advs73950‐sup‐0001‐SuppMat.docx. [file ADVS-9999-e21625-s001.docx]

Supporting Information

Atomic Tuning of Metal-Support Interactions for Pathway-Selective CO_2_ Photoreduction on TiO_2_

Dongyun Kim,^‡^ Wonjae Ko,^‡^ Byoung-Hoon Lee,^‡^ Sanghoon Kim, Yun Do Kim, Hyunsoo Ahn, Yoon Jung, Chan Woo Lee, Kug-Seung Lee, Eunhee Gong, Junho Lee, Minho Kim,* Taeghwan Hyeon,* and Su-Il In*

^[‡]^These authors contributed equally to this work.

D. Kim, E. Gong, J. Lee, S.-I. In

Department of Energy Science & Engineering, DGIST, Daegu 42988, Republic of Korea.

E-mail: insuil@dgist.ac.kr

W. Ko, H. Ahn, Y. Jung, C. W. Lee, T. Hyeon
Center for Nanoparticle Research, Institute for Basic Science (IBS), Seoul 08826, Republic of Korea.

E-mail: thyeon@snu.ac.kr

W. Ko, H. Ahn, Y. Jung, C. W. Lee, T. Hyeon

School of Chemical and Biological Engineering, and Institute of Chemical Processes, Seoul National University, Seoul 08826, Republic of Korea.

B.-H. Lee, Y. D. Kim

KU-KIST Graduate School of Converging Science and Technology, Korea University, Seoul 02841, Republic of Korea

B.-H. Lee

Department of Integrative Energy Engineering, Korea University, Seoul 02841, Republic of Korea

S. Kim, M. Kim

Department of Applied Chemistry, Kyung Hee University, Yongin, Gyeonggi 17104, Republic of Korea.

E-mail: minho.kim@khu.ac.kr

K.-S. Lee

Pohang Accelerator Laboratory (PAL), Pohang 37673, Republic of Korea


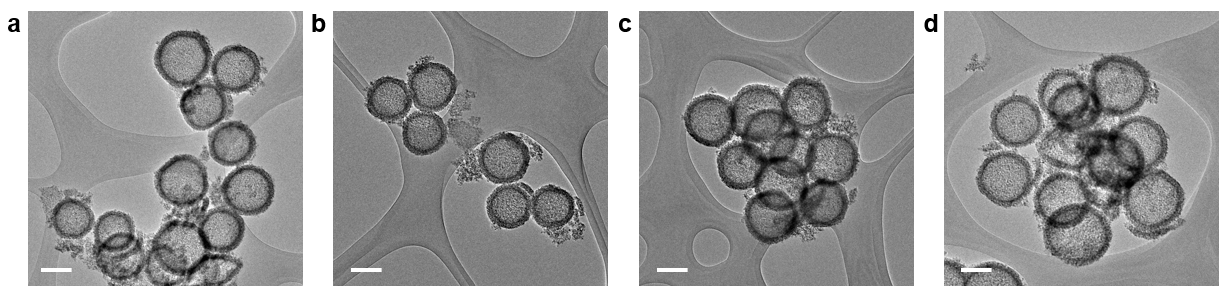


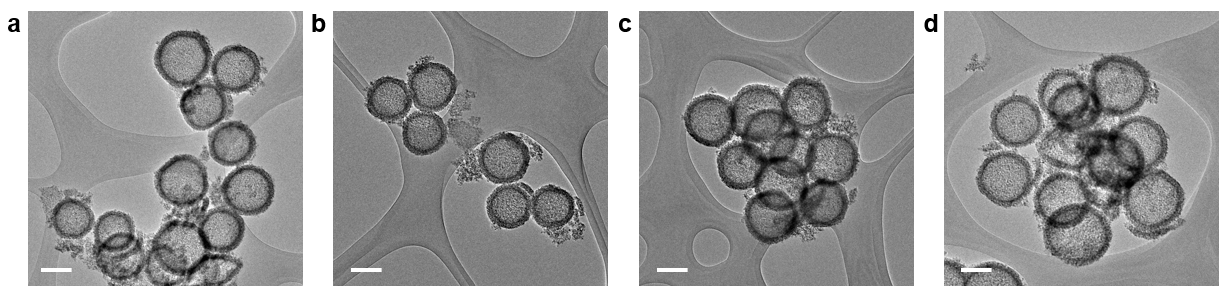


**Figure S1.** TEM images of (a) 0.1Fe/TiO_2_, (b) 0.2Fe/TiO_2_, (c) 0.5Fe/TiO_2_, (d) 1.4Fe/TiO_2_. Scale bars in (a–d) = 200 nm.


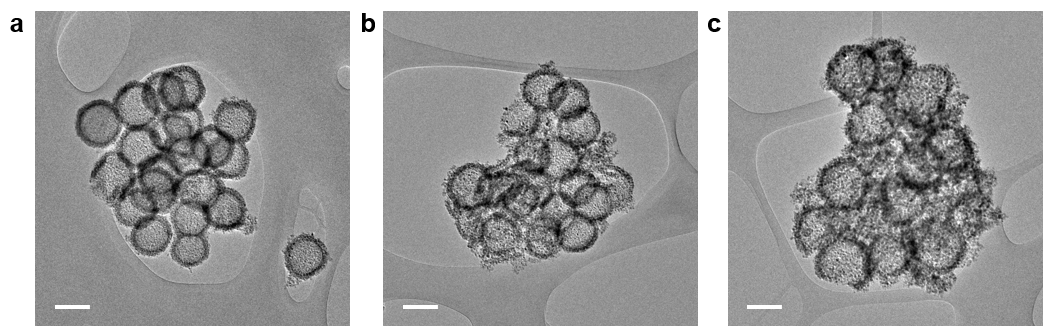


**Figure S2.** TEM images of (a) 0.2Cu/TiO_2_, (b) 0.6Cu/TiO_2_, (c) 1.0Cu/TiO_2_. Scale bars in (a–c) = 200 nm.


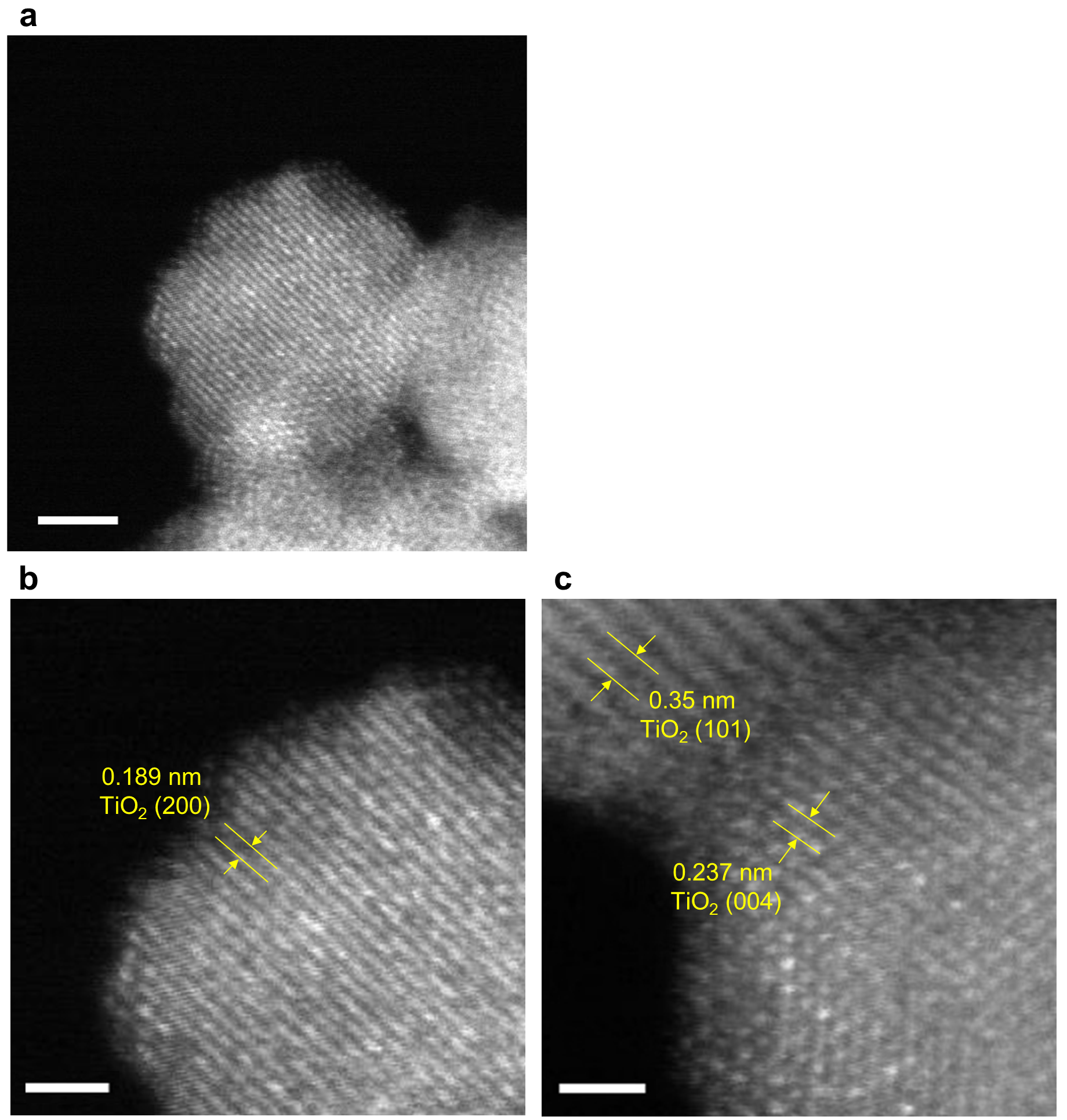


**Figure S3.** (a) HAADF-STEM images of 0.6Cu/TiO_2_. Scale bar = 2 nm. Enlarged high-resolution images extracted from HAADF-STEM micrographs of (b) 0.6Cu/TiO_2_ and (c) 0.5Fe/TiO_2_, where lattice fringes are indexed to anatase TiO_2_ crystal planes. Scale bar = 1 nm.


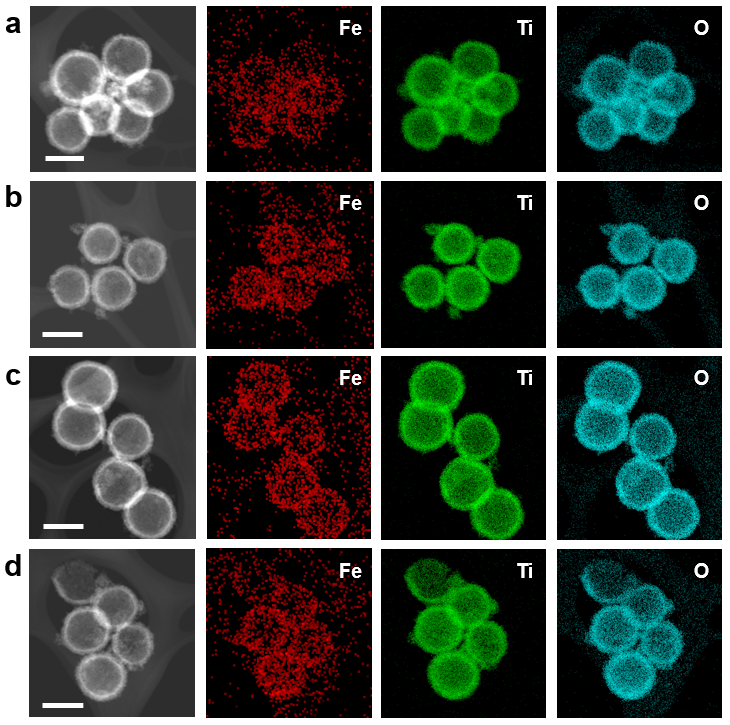


**Figure S4.** EDS elemental mappings of (a) 0.1Fe/TiO_2_, (b) 0.2Fe/TiO_2_, (c) 0.5Fe/TiO_2_, (d) 1.4Fe/TiO_2_. Scale bars in (a–d) = 250 nm.


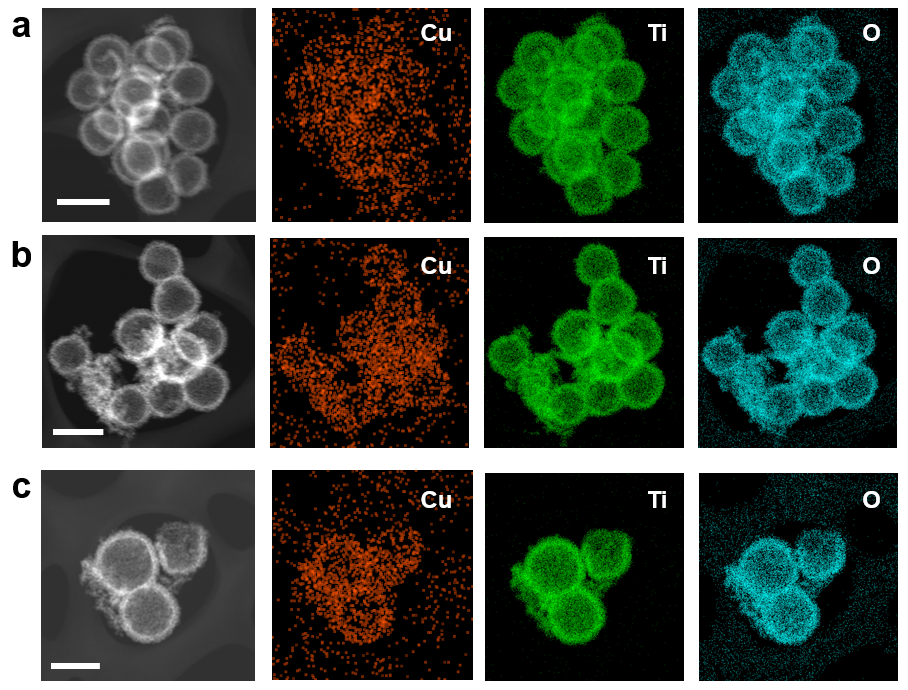


**Figure S5.** EDS elemental mappings of (a) 0.2Cu/TiO_2_, (b) 0.6Cu/TiO_2_, (c) 1.0Cu/TiO_2_. Scale bars in (a–c) = 250 nm.


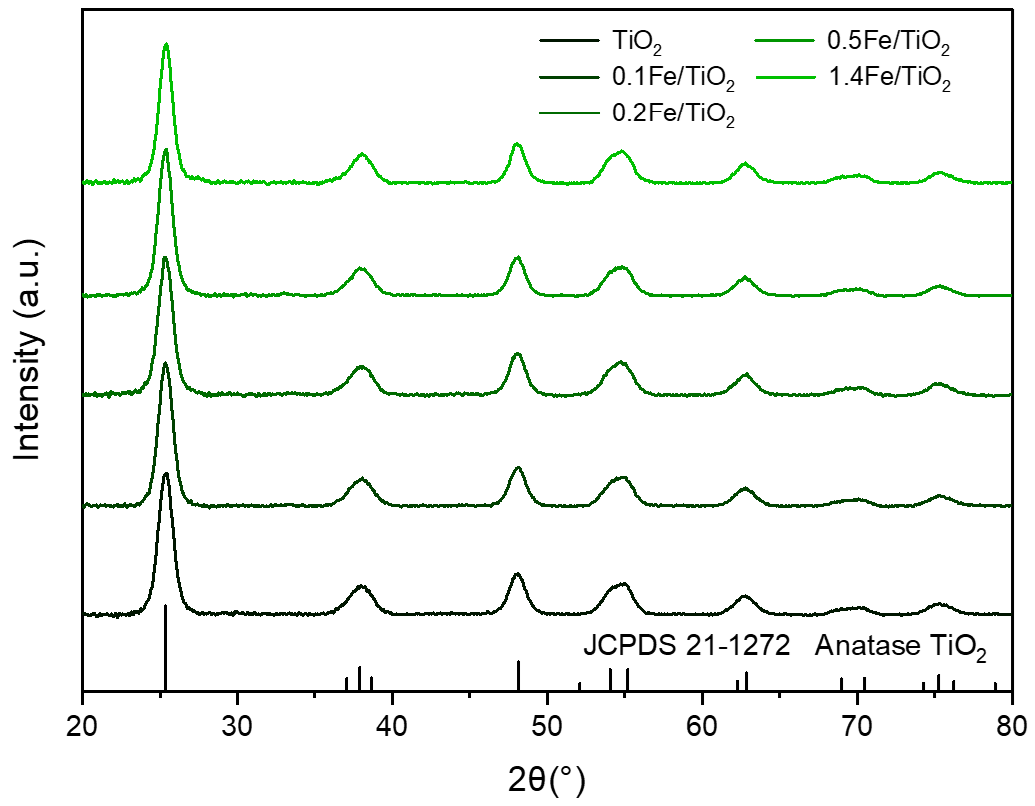


**Figure S6.** XRD patterns of TiO_2_, 0.1Fe/TiO_2_, 0.2Fe/TiO_2_, 0.5Fe/TiO_2_, and 1.4Fe/TiO_2_.


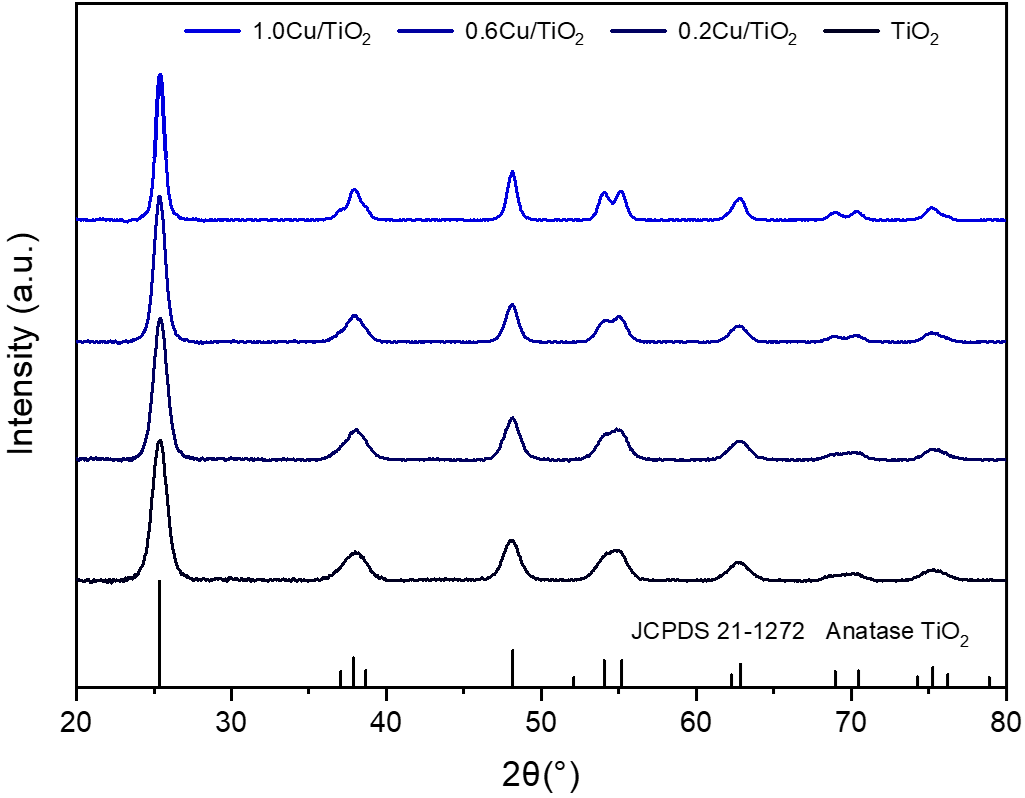


**Figure S7.** XRD patterns of TiO_2_, 0.2Cu/TiO_2_, 0.6Cu/TiO_2_, and 1.0Cu/TiO_2_.


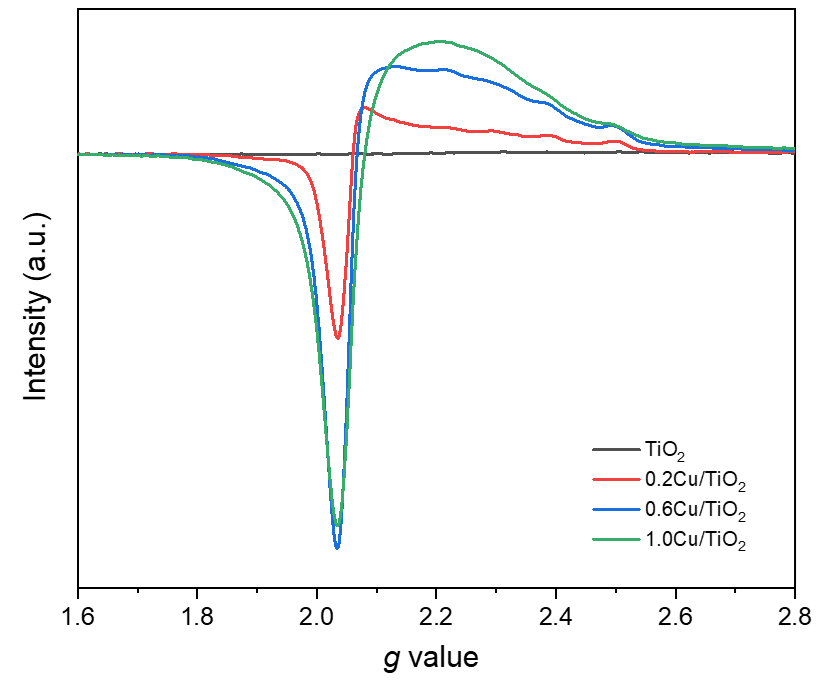


**Figure S8.** EPR spectra of TiO_2_, 0.2Cu/TiO_2_, 0.6Cu/TiO_2_, and 1.0Cu/TiO_2_ measured at 150 K.


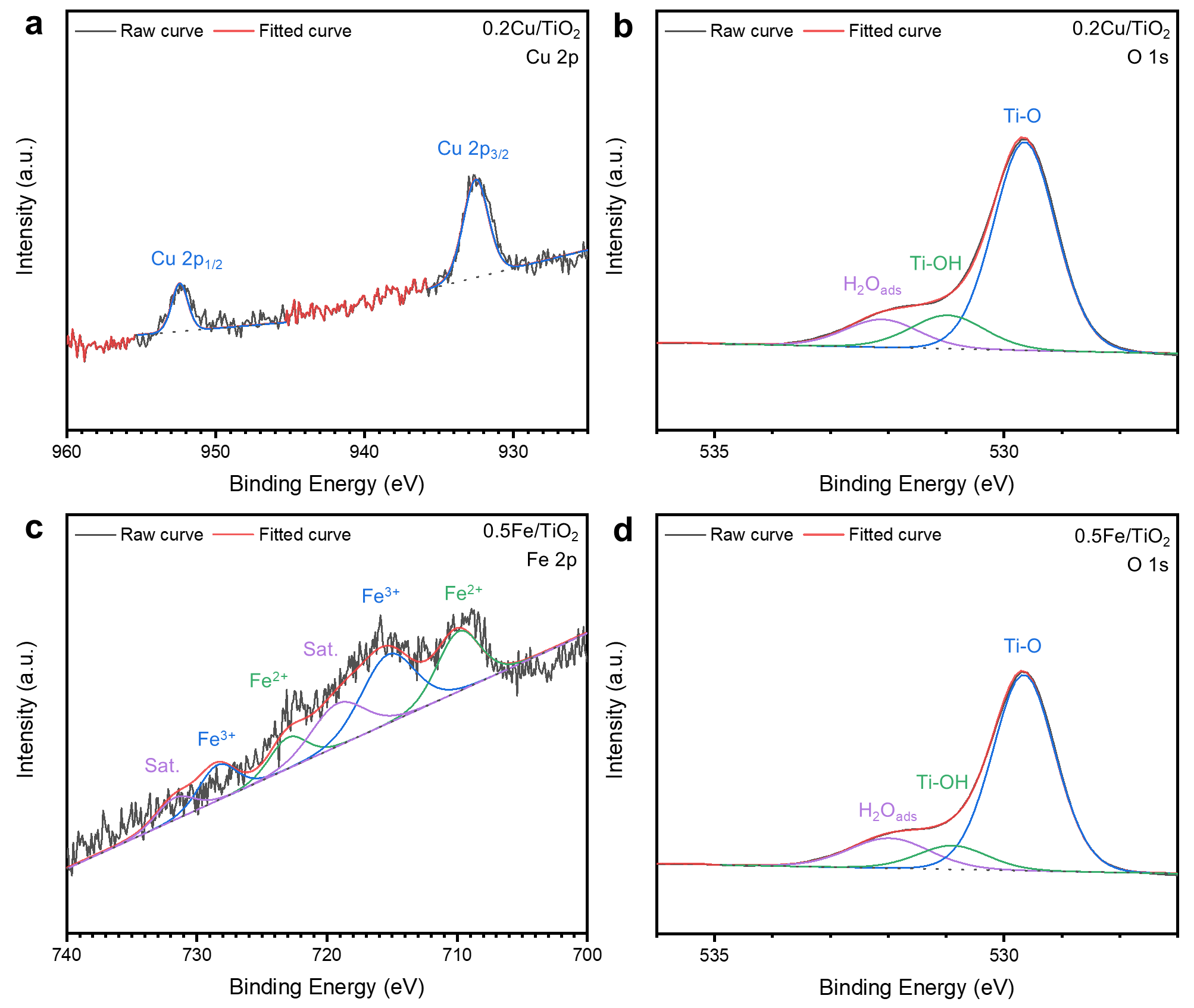


**Figure S9.** XPS spectra of the optimized catalysts. (a) Cu 2p and (b) O 1s spectra of 0.2Cu/TiO_2_, and (c) Fe 2p and (d) O 1s spectra of 0.5Fe/TiO_2_. Sat. denotes satellite peaks.


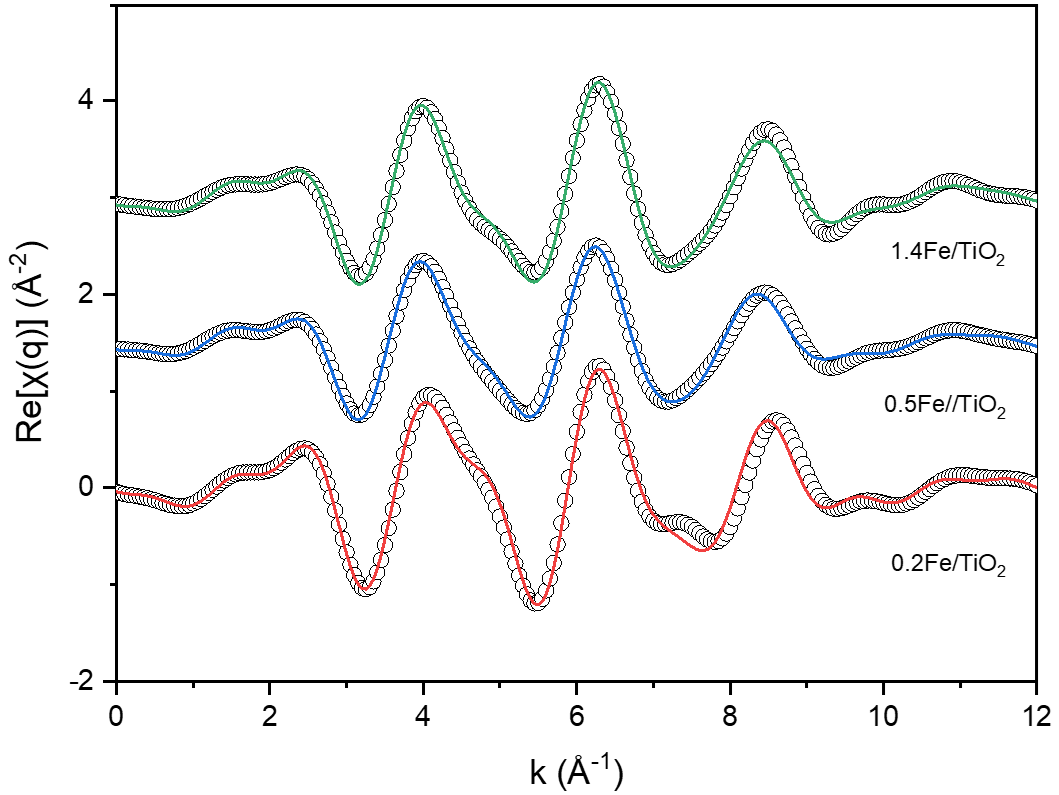


**Figure S10.** Fe *K*-edge EXAFS of 0.2Fe/TiO_2_, 0.5Fe/TiO_2_, 1.4Fe/TiO_2_ in *k*-space (circles), and their fitting results (lines).


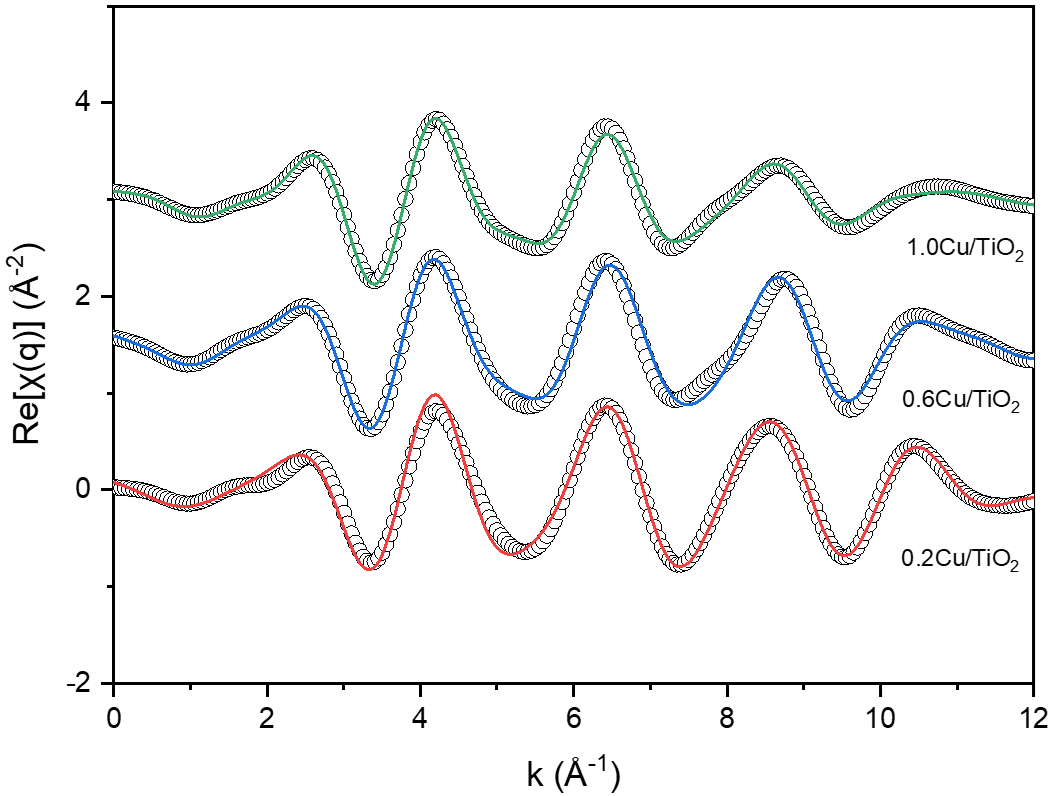


**Figure S11.** Cu *K*-edge EXAFS of 0.2Cu/TiO_2_, 0.6Cu/TiO_2_, 1.0Cu/TiO_2_ in *k*-space (circles), and their fitting results (lines).


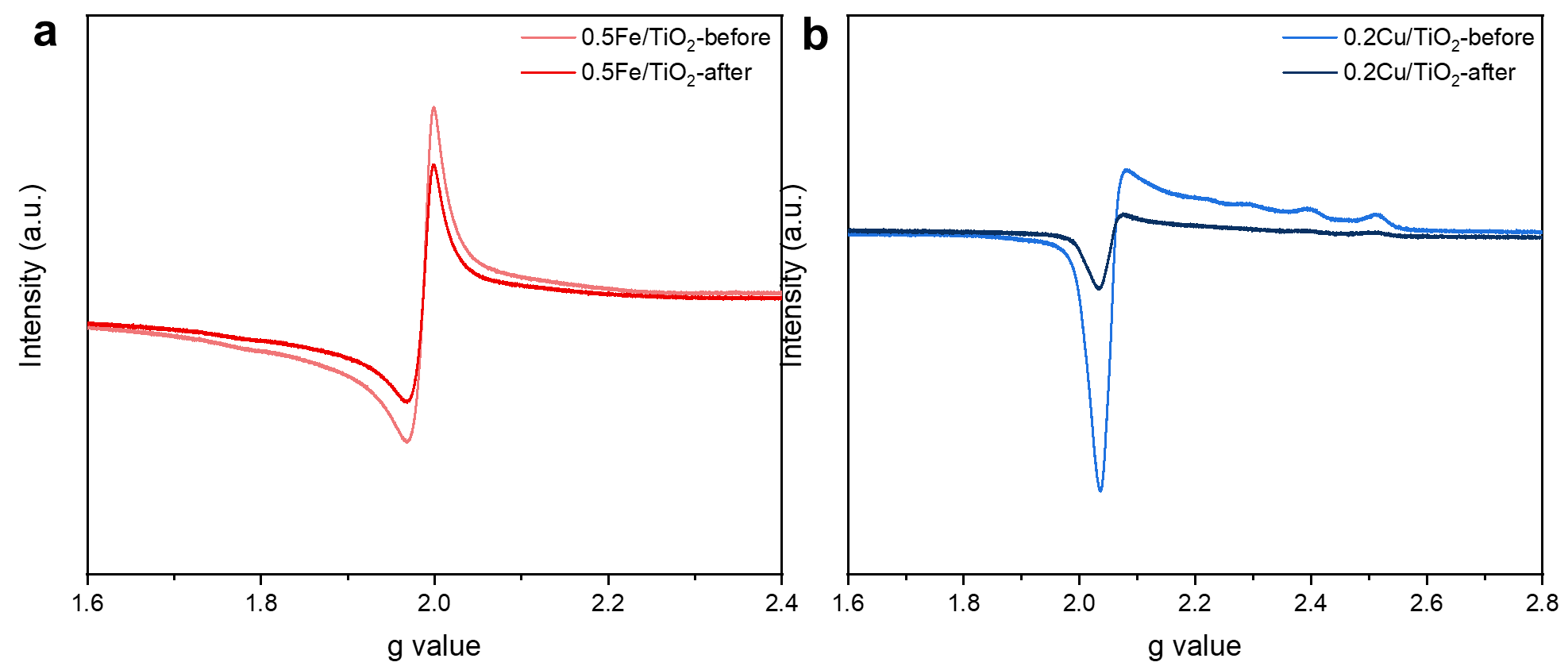


**Figure S12.** EPR spectra before and after photocatalytic irradiation for (a) 0.5Fe/TiO_2_ and (b) 0.2Cu/TiO_2_ measured at 150 K.


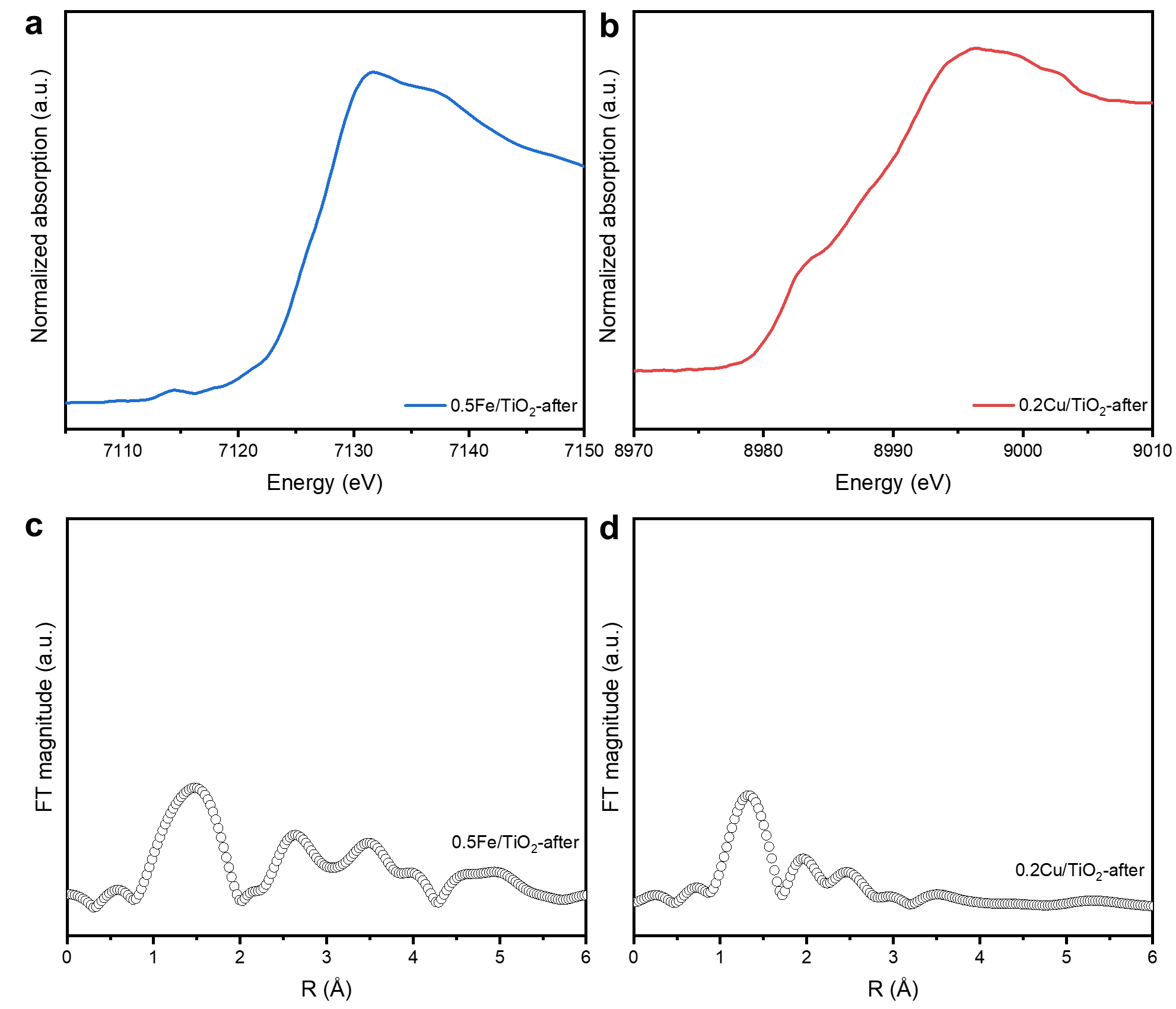


**Figure S13.** (a) Fe K-edge XANES of 0.5Fe/TiO_2_ and (b) Cu K-edge XANES of 0.2Cu/TiO_2_ after photocatalytic irradiation. (c) Fe K-edge of 0.5Fe/TiO_2_ and (d) Cu K-edge FT-EXAFS of 0.2Cu/TiO_2_ after irradiation.


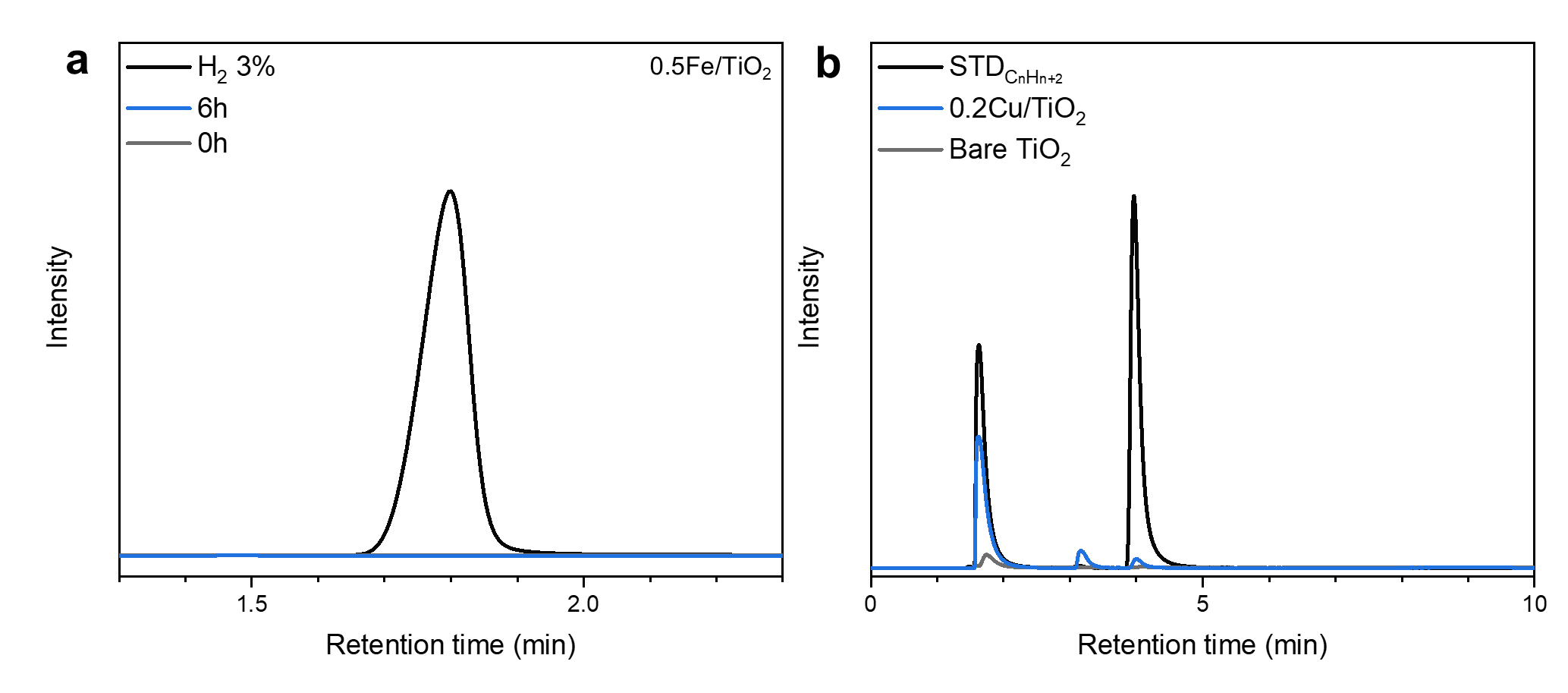


**Figure S14.** Gas chromatograms obtained during CO_2_ reduction after 6 h irradiation, showing (a) hydrogen evolution over 0.5Fe/TiO_2_ and (b) hydrocarbon evolution over 0.2Cu/TiO_2_.


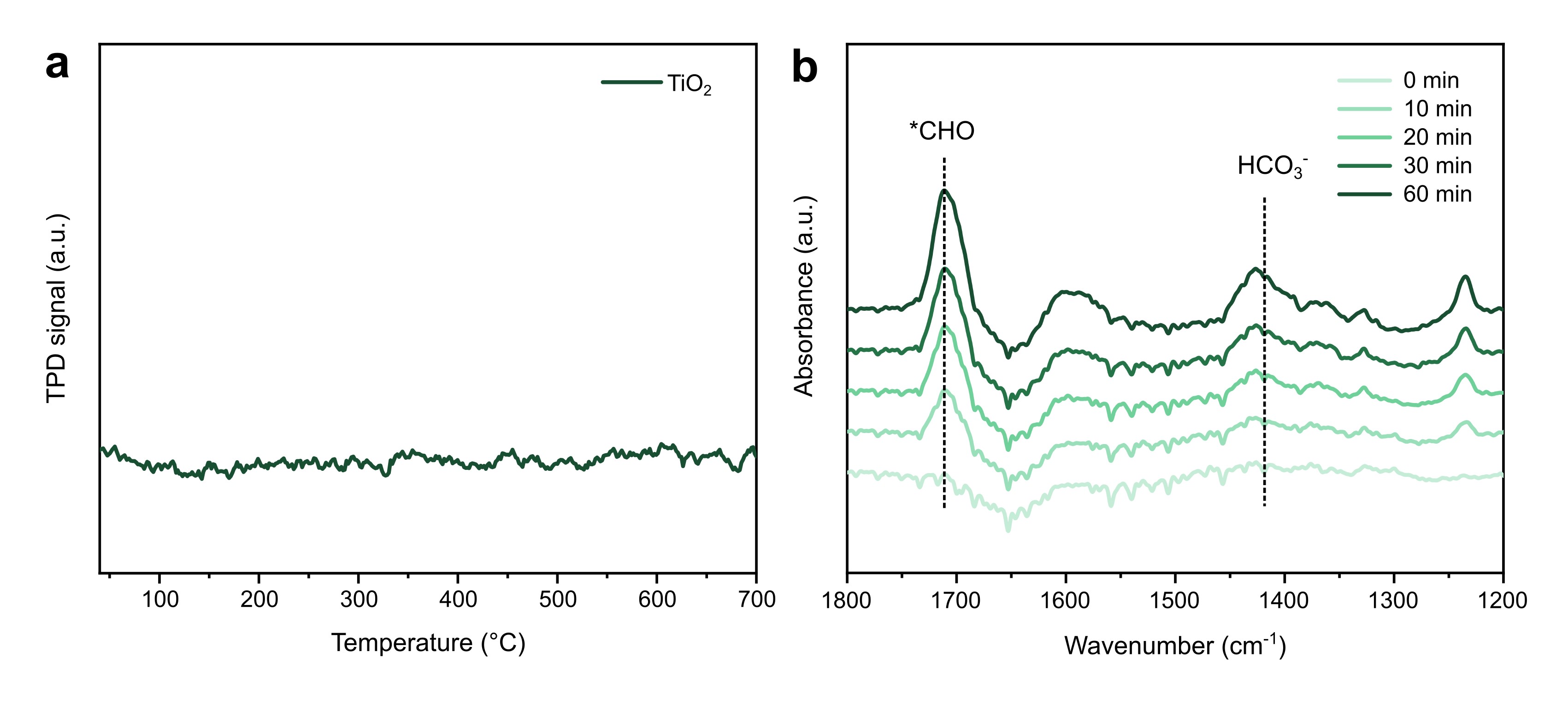


**Figure S15.** (a) TPD analysis and (b) DRIFT analysis of bare TiO_2_.


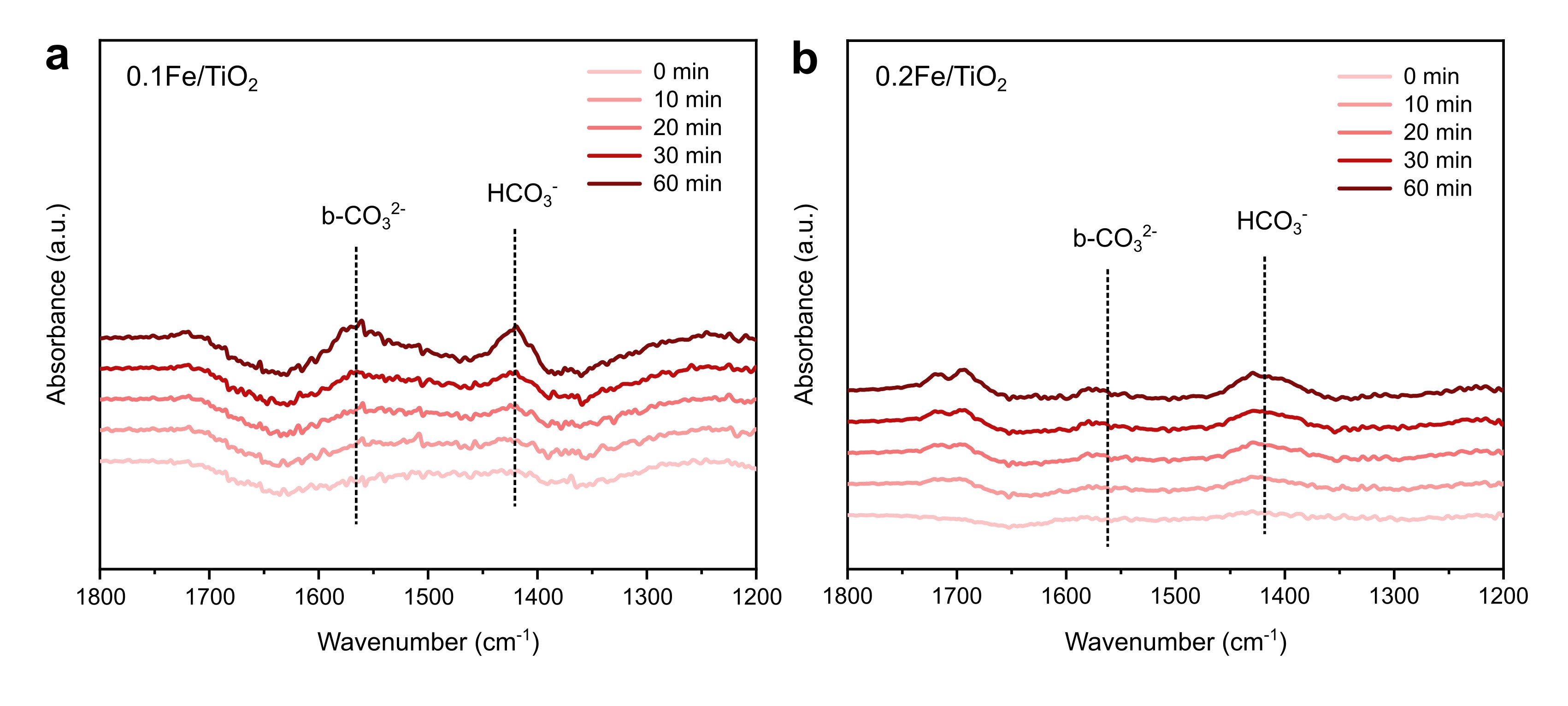


**Figure S16.** DRIFT analysis of (a) 0.1Fe/TiO_2_, (b) 0.2Fe/TiO_2_.


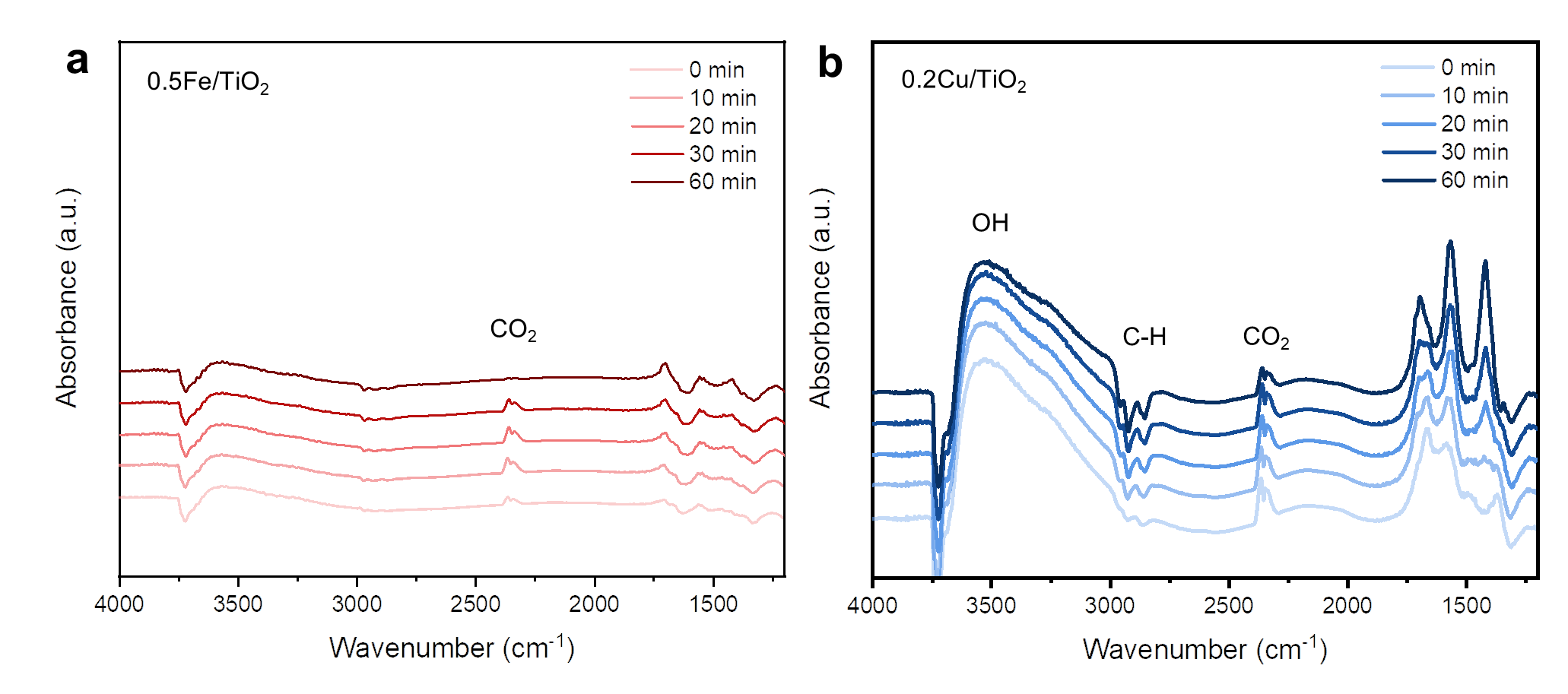


**Figure S17.** DRIFT analysis from 4000 to 1200 cm^-1^ of (a) 0.5Fe/TiO_2_, (b) 0.2Cu/TiO_2_.


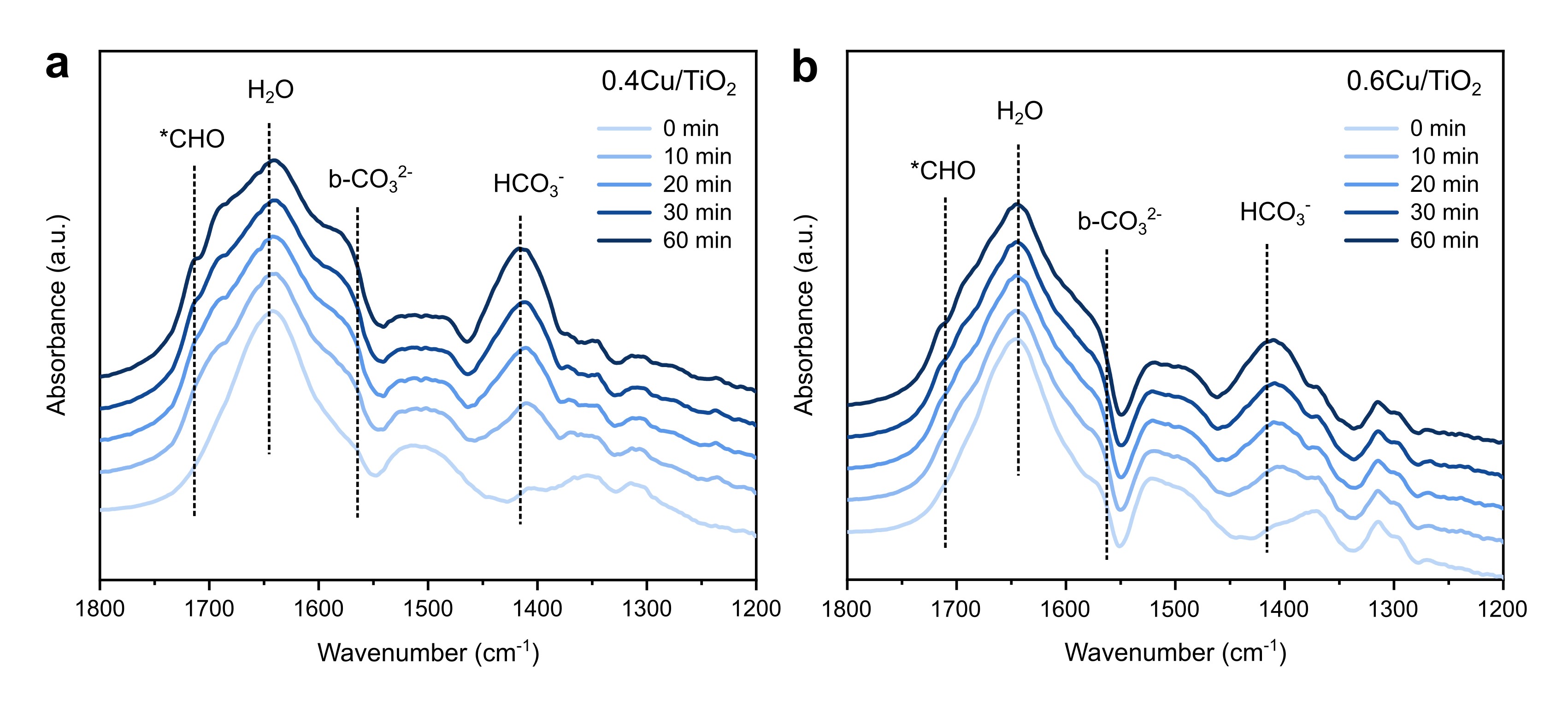


**Figure S18.** DRIFT analysis of (a) 0.4Cu/TiO_2_, (b) 0.6Cu/TiO_2_.


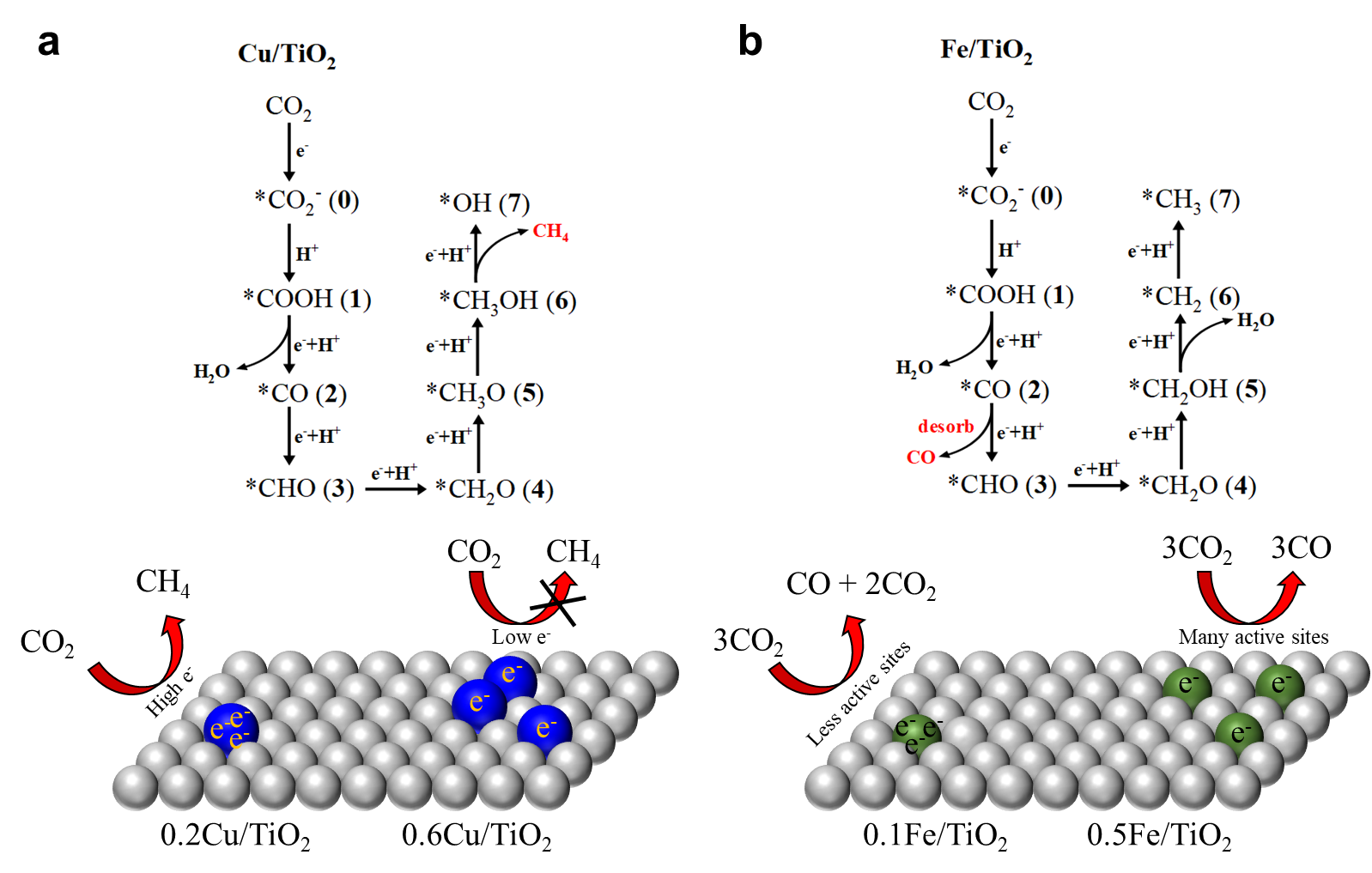


**Figure S19.** CO_2_ reduction reaction in (a) Cu/TiO_2_ and (b) Fe/TiO_2_. The atoms are coloured as gray for Ti, blue for Cu, dark green for Fe.


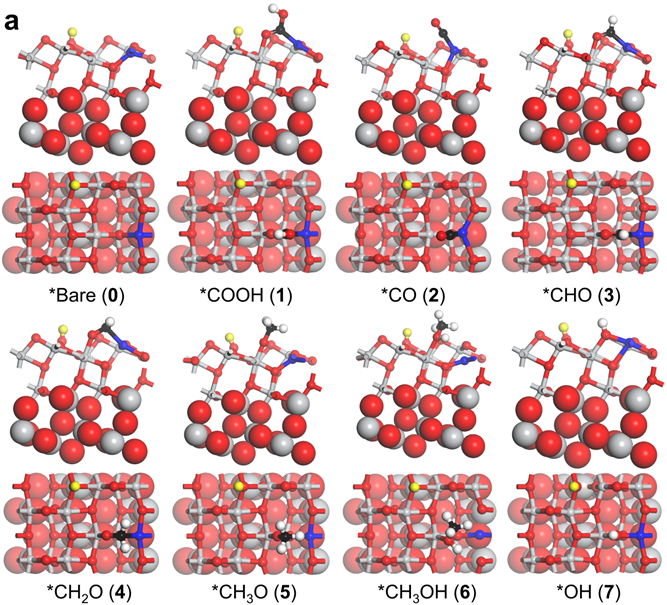


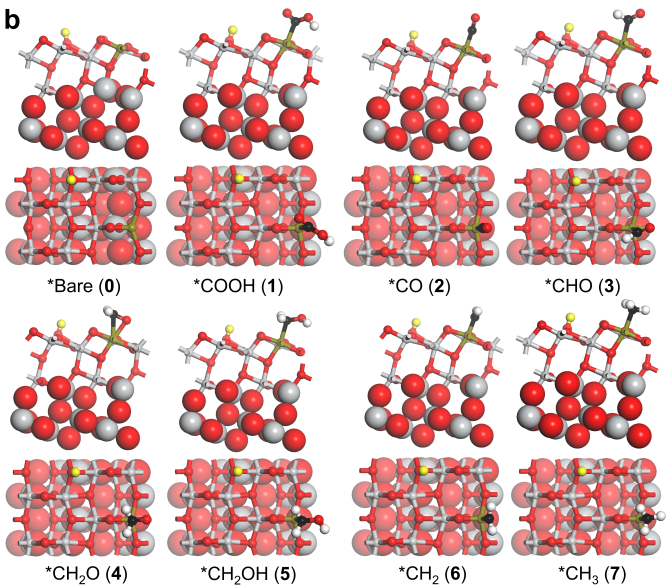


**Figure S20.** DFT optimized structures of CO_2_ reduction intermediates in (a) Cu/TiO_2-x_ and (b) Fe/TiO_2_ from (top) side view and (bottom) top views. The atoms are colored as red for O, gray for Ti, black for C, blue for Cu, dark green for Fe, white for H, and yellow for the hydrogen atom that introduces a photogenerated electron. The bold numbers in parentheses are the number of reduction steps in Figure 4e, and the intermediates at step 5 to 7 differ between Cu/TiO_2-x_ and Fe/TiO_2_ based on the existence of oxygen vacancy as a binding pocket.





**Figure S21.** Local geometry of the metal-support coordination of a single-atom catalyst on TiO_2_. The numbers are the distances between the single-atom metal and lattice O atoms, and the atoms are colored in red for O, gray for Ti, brown for Fe, and blue for Cu, respectively.


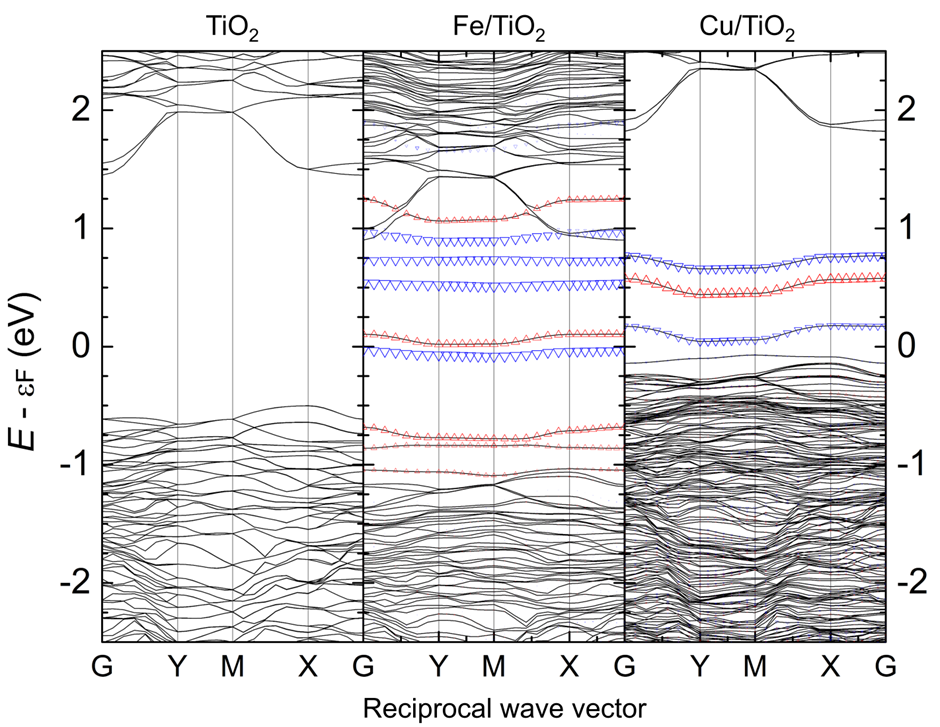


**Figure S22.** DFT band structure of TiO_2_, Fe/TiO_2_ and Cu/TiO_2_ (101) surfaces with the fatband for the single metal atoms (Fe in Fe/TiO_2_ and Cu in Cu/TiO_2_, respectively), where up and down spins are indicated as red up triangles (△) and blue down triangles (▽), respectively.


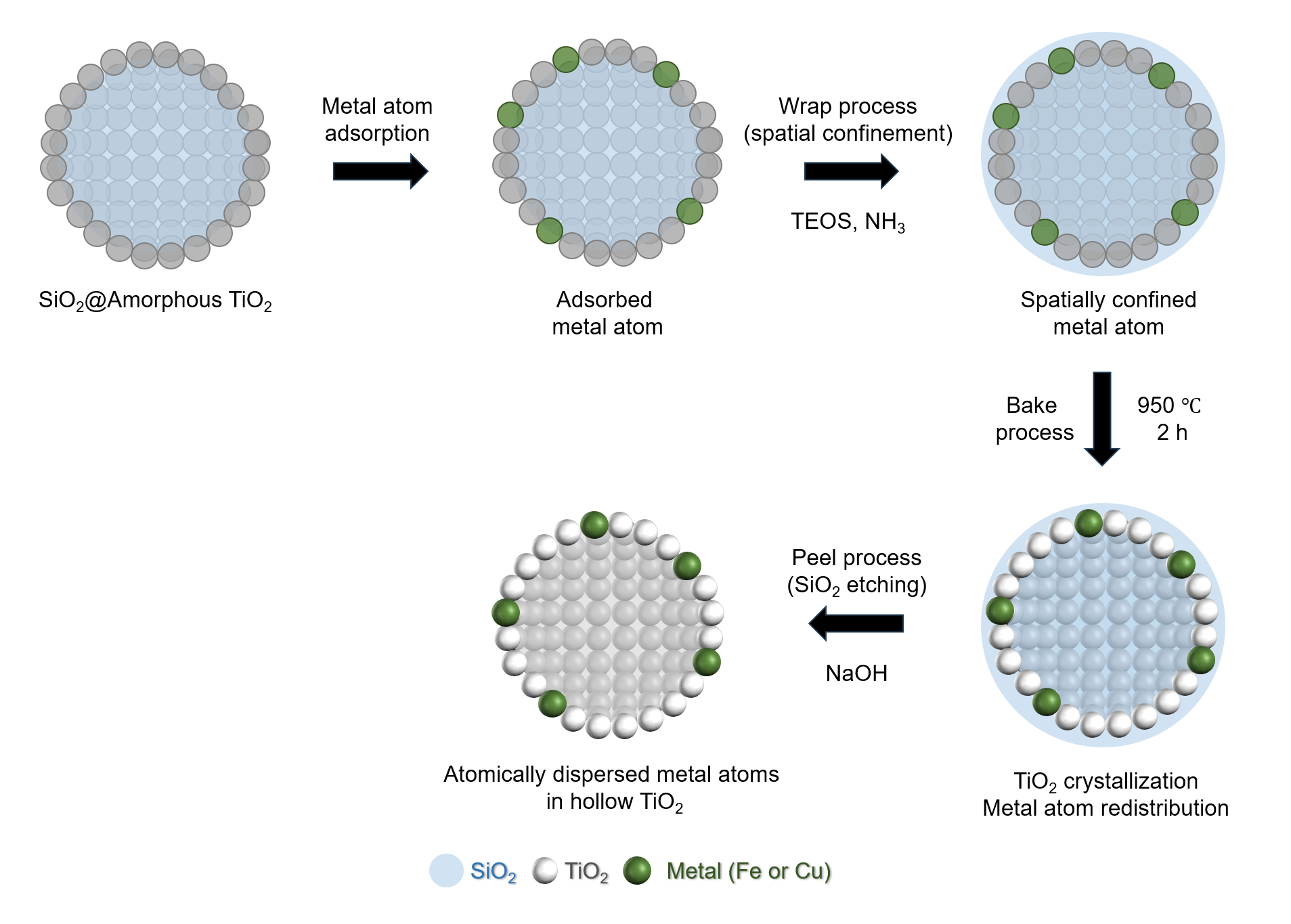


**Figure S23.** Schematic illustration of the wrap–bake–peel process for synthesizing Fe and Cu single atom catalysts anchored on hollow TiO_2_. TEOS denotes tetraethyl orthosilicate.


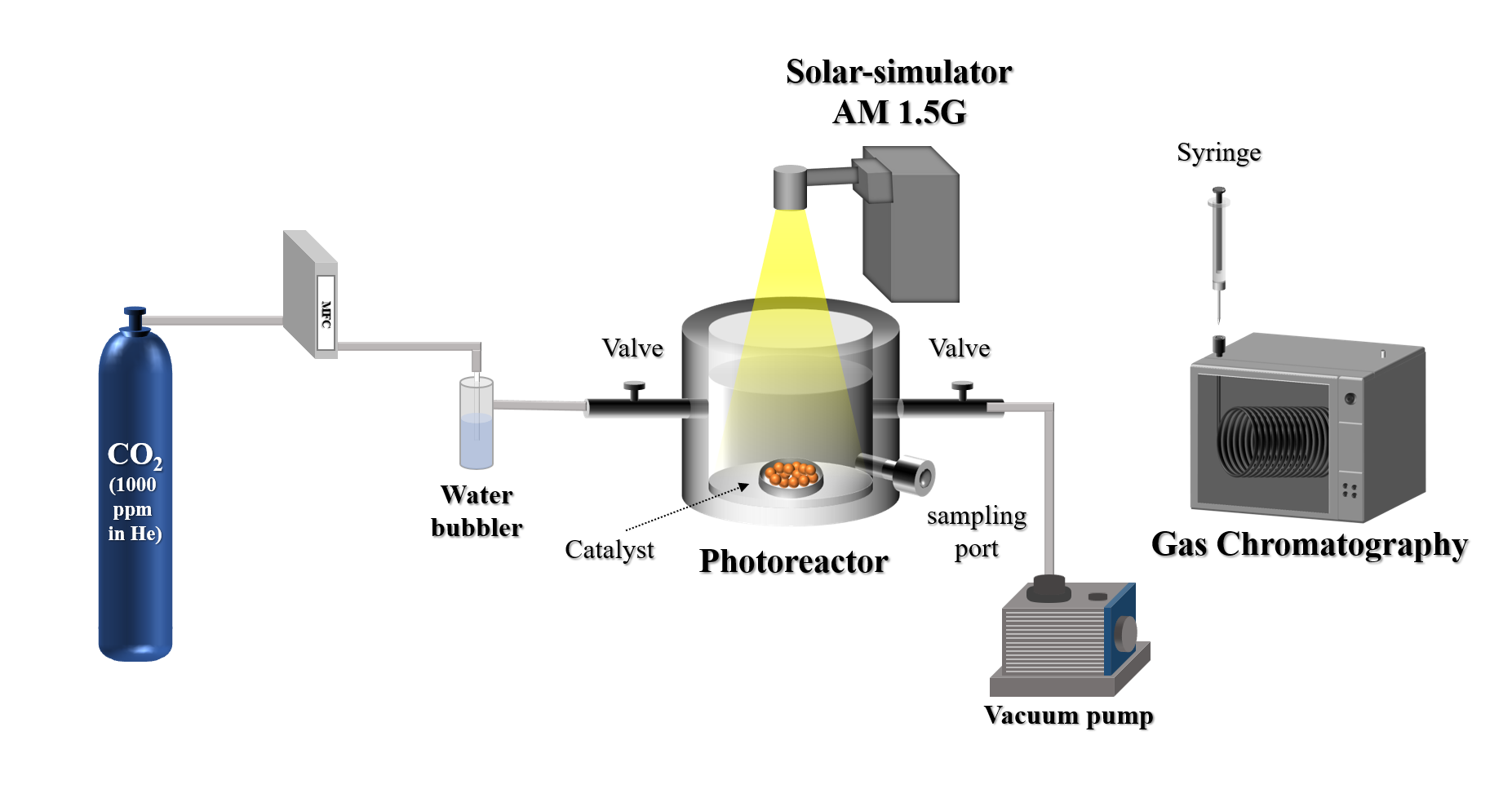


**Figure S24.** Schematic of the photocatalytic CO_2_ reduction system.


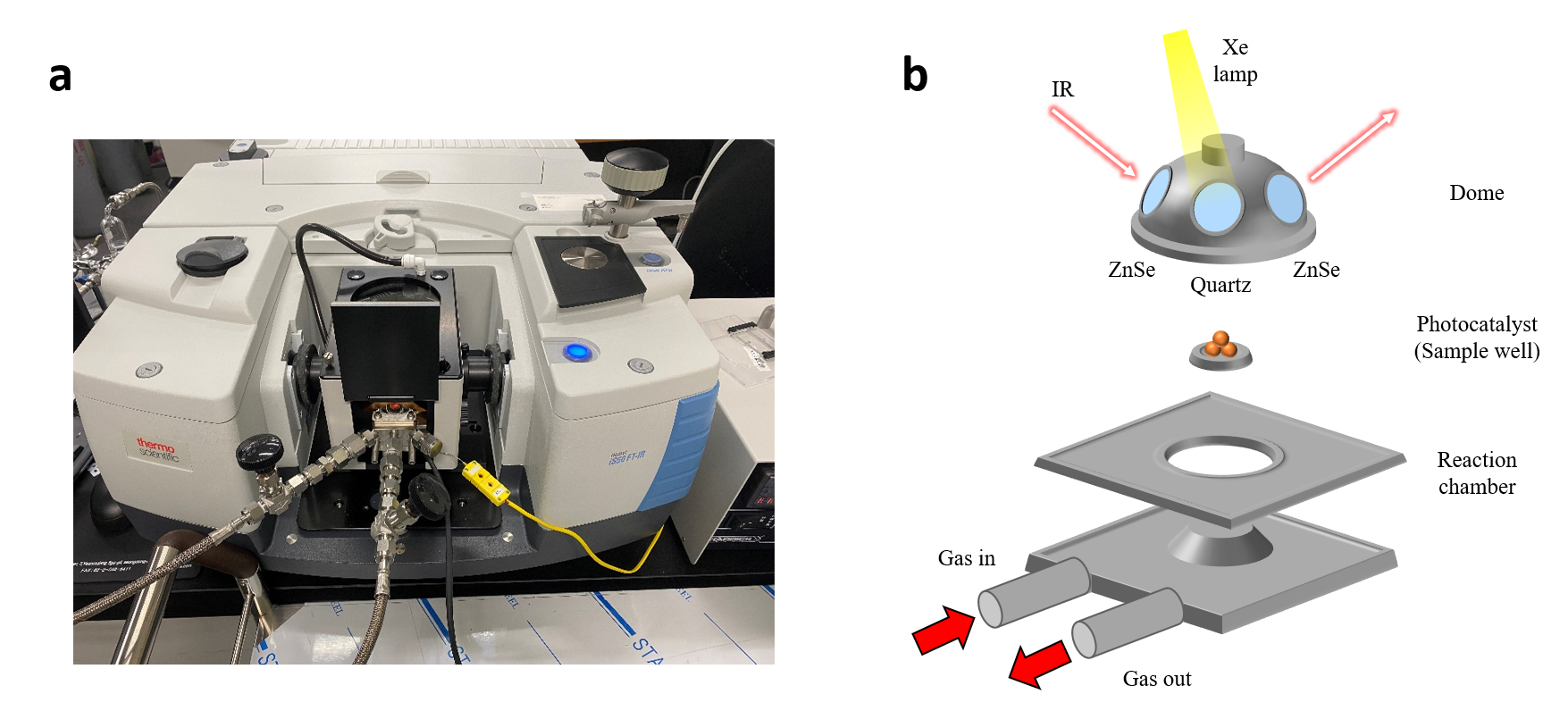


**Figure S25.** Schematic diagram of the DRIFT spectroscopy setup: (a) the entire system and (b) the reactor chamber.

| **Samples** | **Volume of 4 mg/ml FeCl_3_·6H_2_O aqueous solution (mL)** | **Fe loading (wt%)** |
| --- | --- | --- |
| 0.1Fe/TiO_2_ | 0.25 | 0.077 |
| 0.2Fe/TiO_2_ | 0.75 | 0.218 |
| 0.5Fe/TiO_2_ | 1.5 | 0.461 |
| 1.4Fe/TiO_2_ | 3.0 | 1.376 |

**Table S1.** Volume of metal precursor solution used in the synthesis of Fe/TiO_2_ nanoparticles, and the resulting metal contents in Fe/TiO_2_ determined by ICP-AES.

**Table S2.** Volume of metal precursor solution used in the synthesis of Cu/TiO_2_ nanoparticles, and the resulting metal contents in Cu/TiO_2_ determined by ICP-AES.

| **Samples** | **Volume of 4 mg/ml CuCl_2_·2H_2_O aqueous solution (mL)** | **Cu loading (wt%)** |
| --- | --- | --- |
| 0.1Cu/TiO_2_ | 0.1 | 0.080 |
| 0.2Cu/TiO_2_ | 0.25 | 0.198 |
| 0.4Cu/TiO_2_ | 0.5 | 0.426 |
| 0.6Cu/TiO_2_ | 0.75 | 0.580 |
| 1.0Cu/TiO_2_ | 1.5 | 1.020 |

**Table S3.** Summary of Fe *K*-edge EXAFS fitting results of 0.2Fe/TiO_2_, 0.5Fe/TiO_2_, and 1.4Fe/TiO_2_. Theoretical models for EXAFS fitting were constructed by adding shells of atoms around the central Fe atom in anatase TiO_2_ structure. N = coordination number, R = interatomic distance, σ^2^ = Debye-Waller factor.

| **Sample** | **Path** | **N** | **R (Å)** | **σ^2^ (Å^2^)** | **R-factor (%)** |
| --- | --- | --- | --- | --- | --- |
| 0.2Fe/TiO_2_ | Fe–O | 5 | 1.93 ± 0.01 | 0.001 ± 0.001 | 1.4 |
|  | Fe–Ti | 3 | 3.06 ± 0.02 | 0.007 ± 0.004 |  |
| 0.5Fe/TiO_2_ | Fe–O | 5 | 1.95 ± 0.01 | 0.003 ± 0.001 | 1.5 |
|  | Fe–Ti | 3 | 3.04 ± 0.03 | 0.014 ± 0.004 |  |
| 1.4Fe/TiO_2_ | Fe–O | 5 | 1.94 ± 0.01 | 0.001 ± 0.001 | 1.5 |
|  | Fe–Ti | 3 | 3.02 ± 0.03 | 0.010 ± 0.003 |  |

**Table S4.** Summary of Cu *K*-edge EXAFS fitting results of 0.2Cu/TiO_2_, 0.6Cu/TiO_2_, and 1.0Cu/TiO_2_. Theoretical models for EXAFS fitting were constructed by adding shells of atoms around the central Cu atom in anatase TiO_2_ structure. N = coordination number, R = interatomic distance, σ^2^ = Debye-Waller factor.

| **Sample** | **Path** | **N** | **R (Å)** | **σ^2^ (Å^2^)** | **R-factor (%)** |
| --- | --- | --- | --- | --- | --- |
| 0.2Cu/TiO_2_ | Cu–O | 4 | 1.90 ± 0.04 | 0.001 ± 0.001 | 1.5 |
|  | Cu–Ti | 2 | 3.09 ± 0.04 | 0.015 ± 0.005 |  |
| 0.6Cu/TiO_2_ | Cu–O | 4 | 1.87 ± 0.01 | 0.001 ± 0.001 | 1.5 |
|  | Cu–Ti | 2 | 3.08 ± 0.04 | 0.015 ± 0.007 |  |
| 1.0Cu/TiO_2_ | Cu–O | 4 | 1.92 ± 0.05 | 0.006 ± 0.001 | 0.6 |
|  | Cu–Ti | 2 | 3.05 ± 0.02 | 0.014 ± 0.004 |  |

**Table S5.** Photocatalytic CO_2_ reduction performance of TiO_2_-based single-atom cocatalysts

| Catalyst | Test condition | | | | Yield  [g^-1^ h^-1^] | Fold vs.  base cat.^a)^ | Notes |
| --- | --- | --- | --- | --- | --- | --- | --- |
|  | Light source | Reactor [mL] | Cat.  [mg] | System |  |  |  |
| Fe/TiO_2_ | Xenon 100 W  100 mW·cm^-2^ | 15.4 | 40 | Gas | CO 1562.5 ppm | CO 55.7x | This work  (vs. TiO_2_) |
| Cu/TiO_2_ | Xenon 100 W  100 mW·cm^-2^ | 15.4 | 40 | Gas | CH_4_ 1416.9 ppm  C_2_H_6_ 64.19 ppm | CH_4_ 44.5x  C_2_H_6_ - | This work  (vs. TiO_2_) |
| Cu-Ti-V_O_/Ti_0.91_O_2_-SL | Xenon 300 W | 166 | 10 | Liquid | CO 18.6 μmol  C_2_H_4_ 7.6 μmol  C_3_H_8_ 13.8 μmol | CO 0.28x  C_2_H_4_ -  C_3_H_8_ - | ^[1]^  (vs. Ti_0.91_O_2_) |
| Pd-SA/TiO_2_ | Xenon 300 W | 400 | 10 | Gas | CO 56.84 μmol  CH_4_ 1.15 μmol | CO 2.53x  CH_4_ 0.66x | ^[2]^  (vs. TiO_2_) |
| Ag/TiO_2_ | Xenon 300 W | - | 5 | Gas | CO 18.4 μmol  CH_4_ 46.0 μmol | CO 1.16x  CH_4_ 11.5x | ^[3]^  (vs. TiO_2_) |
| Pt-Au/R-TNTs | 365 nm LED  208 mW·cm^-2^ | 15 | - | Gas | CH_4_ 360.0 μmol  C_2_H_6_ 28.8 μmol | CH_4_ 149x  C_2_H_6_ - | ^[4]^  (vs. R-TNT) |
| Cu/TiO_2_ | Xenon 300 W | - | 100 | Liquid | CO ≃1.60 μmol | CO 10.35x | ^[5]^  (vs. TiO_2_) |
| Cu-SAs/TiO_2_ | Xenon 300 W  200 mW·cm^-2^ | 170 | 5 | Gas | CO 13.71 μmol  CH_4_ 23.11 μmol | CO 5.12x  CH_4_ - | ^[6]^  (vs. TiO_2_) |

a) Fold increases are referenced to TiO_2_ reported under identical or comparable conditions within each study.

**References**

[1] Y. Shen, C. Ren, L. Zheng, X. Xu, R. Long, W. Zhang, Y. Yang, Y. Zhang, Y. Yao, H. Chi, J. Wang, Q. Shen, Y. Xiong, Z. Zou, Y. Zhou, Room-temperature photosynthesis of propane from CO_2_ with Cu single atoms on vacancy-rich TiO_2_, *Nat. Commun.* **2023**, *14*, 1117.

[2] Y. Zheng, W. Li, J. Ju, J. Jiang, L. Zhang, H. Jiang, Y. Hu, C. Li, Oxygen vacancy mediated Pd-SA/TiO_2_ single-atom catalyst created via ultra-fast one-step synthesis for enhanced CO_2_ photoreduction, *J. Colloid Interface Sci.* **2025**, *683*, 280.

[3] C. Ban, Y. Wang, Y. Feng, Z. Zhu, Y. Duan, J. Ma, X. Zhang, X. Liu, K. Zhou, H. Zou, D. Yu, X. Tao, L. Gan, G. Han, X. Zhou, Photochromic single atom Ag/TiO_2_ catalysts for selective CO_2_ reduction to CH_4_, *Energy Environ. Sci.* **2024**, *17*, 518.

[4] H. Pan, X. Wang, Z. Xiong, M. Sun, M. Murugananthan, Y. Zhang, Enhanced photocatalytic CO_2_ reduction with defective TiO_2_ nanotubes modified by single-atom binary metal components, *Environ. Res.* **2021**, *198*, 111176.

[5] Z. Jiang, W. Sun, W. Miao, Z. Yuan, G. Yang, F. Kong, T. Yan, J. Chen, B. Huang, C. An, G. A. Ozin, Living Atomically Dispersed Cu Ultrathin TiO2 Nanosheet CO_2_ Reduction Photocatalyst, *Adv. Sci.* **2019**, *6*, 1900289.

[6] T. Wang, F. Sun, S. Liu, G. Zhuang, B. Li, Dioxygen-enhanced CO_2_ photoreduction on TiO_2_ supported Cu single-atom sites, *Appl. Catal. B-Environ.* **2023**, *325*, 122339.
